# Reading between the Chains: Surface Mapping and Druggable Pockets on the Biological Assemblies of DENV-2’s Protein E

**DOI:** 10.1021/acsomega.5c08709

**Published:** 2026-01-26

**Authors:** Pedro T.T. F. Leite, Philipe O. Fernandes, Pedro S. Lacerda, Marcelo A. Chagas, Marcelo S. Castilho, Willian R. Rocha, Adolfo Henrique Moraes, Vinícius G. Maltarollo

**Affiliations:** † Departamento de Produtos Farmacêuticos, Faculdade de Farmácia, Universidade Federal de Minas Gerais (UFMG), Av. Antônio Carlos 6627, Belo Horizonte, Minas Gerais 31270-901, Brasil; ‡ Programa de Pós-Graduação Em Farmácia, Faculdade de Farmácia, Universidade Federal da Bahia (UFBA), R. Barão de Jeremoabo s/n, Ondina, Salvador, Bahia 40170-115, Brasil; § Departamento de Ciências Exatas, 133635 Universidade Do Estado de Minas Gerais (UEMG), Av. Brasília, 1304, João Monlevade, Minas Gerais 35930-314, Brasil; ∥ Departamento de Química, Instituto de Ciências Exatas, 28114 Universidade Federal de Minas Gerais (UFMG), Av. Antônio Carlos 6627, Belo Horizonte, Minas Gerais 31270-901, Brasil; ⊥ Laboratório de Ressonância Magnética Nuclear (LAREMAR), Universidade Federal de Minas Gerais (UFMG), Av. Antônio Carlos 6627, Belo Horizonte, Minas Gerais 31270-901, Brasil

## Abstract

Dengue is a widespread arboviral infection endemic to tropical and subtropical regions, representing a major global public health concern. Although most prevalent in South America and Asia, cases have also emerged in Europe and the United States, including instances of local transmission and fatalities. The envelope protein (protein E) is a critical structural component of the viral surface and a well-established molecular target for antiviral drug discovery, owing to its essential role in viral entry. While previous studies have primarily focused on the β-OG pocket within isolated protein E dimers, the mature virion features a complex icosahedral organization composed of protein E dimers adopting distinct assemblies, which might expose novel pockets not present in the dimeric forms, offering new opportunities for structure-based drug design. In this study, we employed molecular dynamics simulations and structure-based computational analyses to identify and characterize druggable pockets on the external surface of protein E in its native oligomeric states. Side chain conformational sampling revealed distinct side chain dynamics and enabled the selection of representative structures for pocket mapping. Several druggable and borderline druggable pockets were identified, including sites encompassing residues such as Lys291, Lys295, and Pro384—key mediators of protein E interaction with host membrane receptors. Additionally, residues such as Glu172, Thr180, Thr303, and Glu383 were found within druggable pockets, supporting their potential inclusion in pharmacophore models. These findings offer valuable insights for pharmacophore modeling and drug repurposing initiatives.

## Graphical abstract

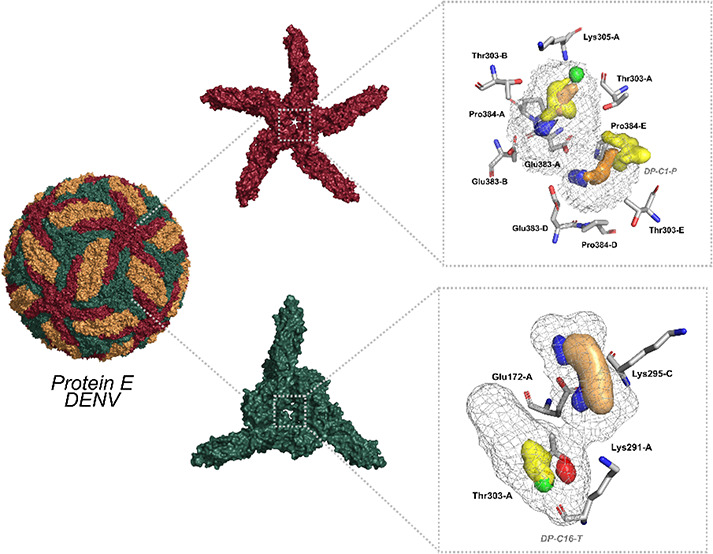

## Introduction

Dengue is an arboviral infection endemic to tropical and subtropical regions, presenting a significant public health challenge. Globally, it accounts for an estimated 390 million new infections annually, with approximately 22,000 deaths.^1,2^ This neglected tropical disease is predominantly concentrated in South America and Asia.^3^ Brazil, for instance, has witnessed an alarming surge, with over 6.6 million estimated cases and six thousand confirmed deaths in 2024 alone, marking the largest outbreak to date.^4^ Dengue cases have also been reported in Europe and the United States, including local transmission in the US and a reported fatality in Florida.^5–8^

Besides the increasing prevalence and worldwide spread, treatment remains symptomatic due to the absence of specific antiviral therapies for the disease. Although dengue vaccine development has shown improvements, the currently licensed CYD-TDV (Dengvaxia) has demonstrated partial efficacy in children and seronegative adults, where there may be an increased risk of severe dengue due to antibody-dependent enhancement.^9,10^ Other candidates, such as TAK-003, have demonstrated promising results on phase III trials for different serotypes.^11^ Recent literature reviews conclude that there is a need for multiple efforts across disciplines and regions to deal with dengue, including antiviral therapy, supportive care, and preventive measures.^10^

Developing effective antivirals depends on identifying molecular targets that are essential for infection and are also structurally druggable.^12^ A deeper understanding of DENV molecular mechanisms, particularly those involved in viral entry, fusion, and early infection events, is therefore crucial.^13^ Current research has proposed several viral targets, including proteases and structural proteins,^14,15^ that can be exploited for antiviral strategies.

Among them, Protein E (protein E), the primary component of the virion surface, plays a central role in viral entry.^15,16^ Structurally, it comprises three domains (DI, DII, and DIII), with DIII being particularly significant for receptor engagement and for harboring neutralizing epitopes that can be exploited for vaccine and therapeutic development.^17–19^ Protein E also mediates interaction with glycosaminoglycans (GAGs) on host-cell surfaces, processes that involve residues located prominently at the oligomeric interfaces of the viral particle.^19^

Previous studies have utilized virtual screening protocols to identify potential ligands in the β-OG pocket between domains I and II^20,21^ (Figure 1). Furthermore, in vitro assays of small molecules against different flaviviruses have suggested that the equivalent pocket is conserved in West Nile, Japanese Encephalitis, Zika Viruses, and DENV.^22^ Conversely, two studies, employing synthetic peptides and thioaptamers, have focused on blocking DIII biological activity.^23,24^ More recently, benzene-mapping studies also suggest that the α pocket, located between DI and DIII, is a suitable target on protein E dimer.^25^ These efforts, however, have concentrated primarily on monomeric and dimeric forms of protein E.

**Figure 1:**
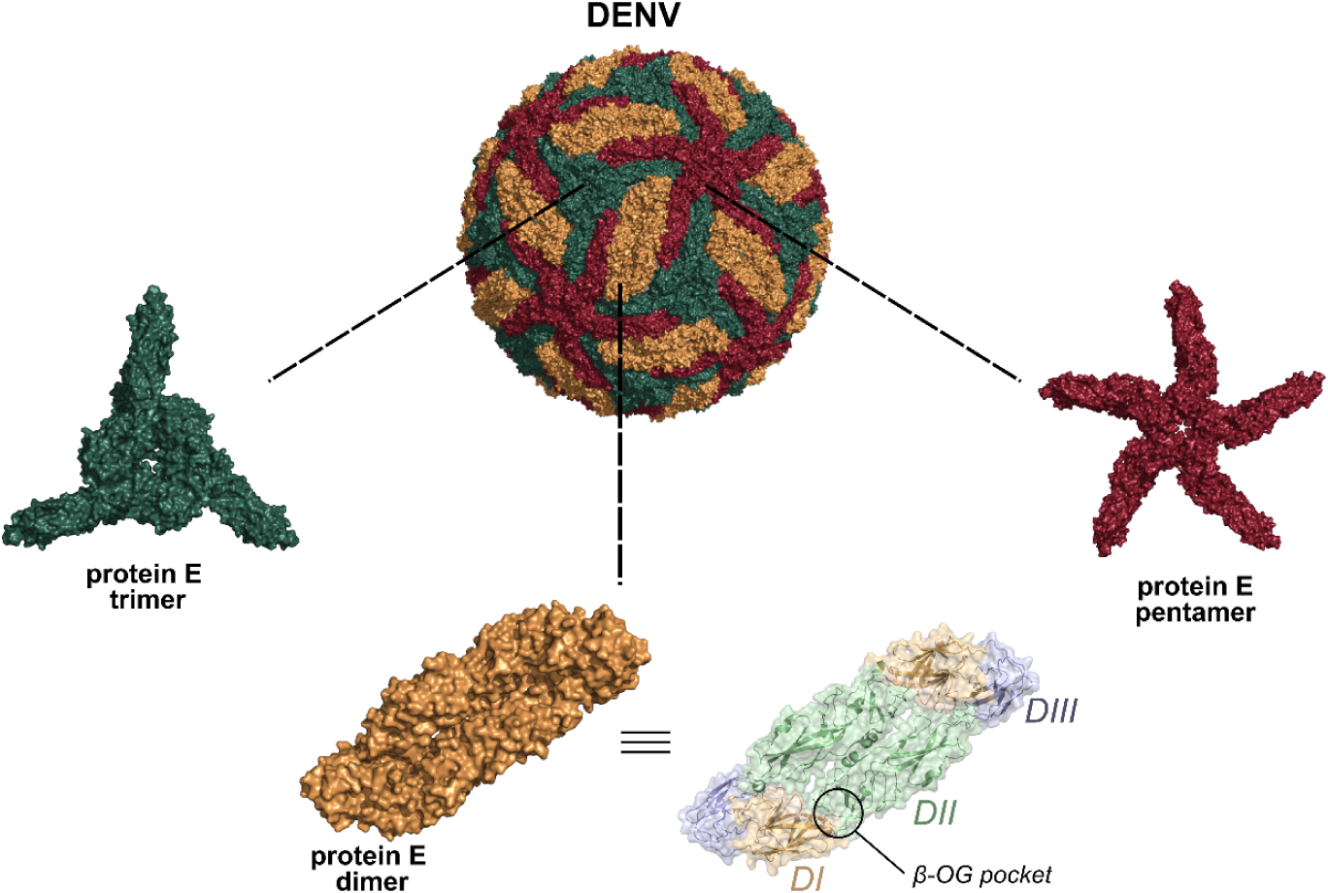
Biological assemblies of protein E in the Dengue virus (DENV) particle. It forms an icosahedral structure, revealing pockets not seen in the isolated protein E dimer (wheat protein surface). These pockets are located in the trimeric (dark green protein surface) and pentameric (dark red protein surface) regions. Cryo-EM structure from PDB ID 3J27.

In the mature virion, protein E adopts a more complex arrangement, forming an icosahedral surface composed of 180 protein E monomers organized into trimeric (3-fold) and pentameric (5-fold) assemblies (Figures 1 and S1). These oligomeric assemblies are not merely geometric features; they correspond to biologically active host-interaction surfaces. Residues implicated in glycosaminoglycan (GAG) binding and heparan sulfate engagement, including Lys291 and Lys295 are displayed prominently at these interfacial regions.^19,26,27^ Several potent neutralizing antibodies also target epitopes at these oligomeric interfaces, highlighting their role as functional determinants of infection rather than as passive structural units.^28,29^ The importance of these surfaces is further supported by the high backbone conservation of trimeric and pentameric assemblies across multiple dengue and flavivirus cryo-EM structures, as we demonstrate in Figure S1.

A major challenge in analyzing cryo-EM structures of DENV is the limited resolution of side chain atoms (typically 2.5–7.0̊Å) which complicates the identification of druggable pockets and accurate modeling of interaction environments^30,31^ Because pocket geometry and hydrogen-bond networks are sensitive to side chain placement, refinement is essential before performing fragment-mapping or ligand-based analyses.^31,32^ Short-time scale molecular dynamics (MD) simulations with backbone restraints, widely used to improve cryo-EM models, provide an effective means of relaxing side chains while preserving the experimentally determined oligomeric scaffolds. The general approach of fixing the backbone and allowing only the side chains to move is a computational technique for sampling rotameric states, reducing simulation cost, and focusing on local interactions and side chain flexibility.^33–35^

In this study, we examine the native trimeric and pentameric assemblies of DENV-2 protein E to identify druggable pockets that emerge exclusively in the context of the viral particle. Using restrained MD to refine uncertain side chain orientations, followed by ensemble-based solvent mapping (FTMap, E-FTMap, and XDrugPy), we characterize hotspot architecture, interaction fingerprints, and pharmacophoric features. Sequence conservation analysis across flaviviruses further illuminates the potential breadth or serotype selectivity of these newly identified pockets. Our findings uncover previously uncharacterized binding sites on the virion surface and provide a framework for exploiting oligomer-specific features of protein E to guide structure-based antiviral design.

## Methods

### Protein Structure Selection

The Protein Data Bank (PDB) was searched using “Dengue virus” and “Electron microscopy” as filters to identify experimentally determined structures of mature DENV particles. The cryo-EM model with PDB ID 3J27,^36^ resolved at 3.6 Å, was selected due to its completeness and high structural similarity to more recent reconstructions (see Table S1 for comparison).

From this structure, the trimeric (3-fold) and pentameric (5-fold) assemblies were extracted (Figure 1) since these oligomeric arrangements represent important native host-interaction surfaces of the mature virion. These interfaces display residues known to mediate GAG binding and receptor-interaction functions, including Lys291 and Lys295. They also correspond to regions targeted by neutralizing antibodies.

Importantly, structural superposition of multiple dengue and flavivirus cryo-EM models (Figure S1) demonstrates that the backbone architecture of these 3-fold and 5-fold assemblies is similar across many structures.

### Rationale for Restrained Molecular Dynamics Simulations

Cryo-EM reconstructions at ∼3–4 Å resolution typically exhibit significant uncertainty in side chain positions, especially in flexible or highly charged residues.^32,37^ Because solvent mapping and pharmacophore identification rely heavily on accurate local geometry, side chain refinement is necessary before conducting fragment-based analyses. To address experimental ambiguity while preserving the native virion architecture, short all-atom MD simulations were performed with harmonic restraints applied to backbone atoms. This approach is well established in cryo-EM model preparation, including flexible fitting and receptor optimization for docking, where backbone restraints maintain the experimental fold while allowing local side chain relaxation to relieve steric strain and improve stereochemical quality.^33–35^

It is worth mentioning that this protocol is not intended to sample global conformational rearrangements or dynamic events. Instead, it ensures a physically reasonable side chain ensemble suitable for subsequent pocket characterization.

### Molecular Dynamics Simulations

All-atom MD simulations were performed for both the trimer and the pentamer with AMBER16 software,^38^ using the ff14SB force field.^39^ Each system was solvated with the TIP3P water model^40^ in a rectangular box with minimal distances of 10 Å between the box edges and any atom. Cl^–^ ions were added to neutralize the system’s net charge. The MD pipeline consisted of two minimization processes: heating and equilibration, followed by production. Both minimization processes comprised 5,000 steps, applying a restriction potential of 100 kcal mol^–1^ Å^–2^.^41^ The first thousand steps were performed using the steepest descent algorithm.^42^ In the remaining steps, the conjugate gradient algorithm was used.^41,42^ The difference between them was the restriction potential applied; the first was employed for all protein atoms, while the second was used only for the protein backbone. Moreover, the backbone restriction was kept during the remaining simulation. The system was heated from 5 to 300 K in the NVT ensemble, alternating heating and equilibration steps. A 50 K temperature increase was applied over 10 ps, followed by 100 ps of equilibration until the final temperature was reached, resulting in a total of six heating steps. At the final temperature, the system was equilibrated for another 5,000 ps. In this ensemble, the Langevin Thermostat^43^ was used to model noncovalent bonds, with collision rates of 2.0 ps^–1^ and a cutoff of 10 Å. Long-range and electrostatic interactions were treated using the particle mesh Ewald (PME) method.^44^ The production was performed on the NPT ensemble at 300 K and 1 bar using the Berendsen isotropic barostat,^45^ applying periodic boundary conditions and a coupling constant of 1.0 ps. The SHAKE algorithm^46^ was employed in both NVT and NPT ensembles to restrict bonds involving hydrogen atoms and reduce high-frequency and dynamic movements. The production stage generated 10 ns trajectories for each system, and their analysis was conducted using only the protein atoms.

Side chain root-mean-square deviation (RMSD) and fluctuation (RMSF) analyses were used only to suggest side chain rotamer convergence, not to infer global dynamic stability, due to the backbone restraints.

### Side Chain Fluctuation and Structural Clustering

The MD data were analyzed with Visual Molecular Dynamics (VMD) 1.9.4 software^47^ to evaluate side chain fluctuation via RMSD. The trajectories were aligned to their first frame, with RMSD analyses performed for three distinct atom selections: first for all protein atoms, then for the central residue in each system (Lys291 in the trimer and Glu383 in the pentamer), and finally for trimmed systems comprising specific residues: Pro39, Thr171-Val181, Lys291-Tyr299, Ser331-Lys334, and Asn355-Thr359 in the trimer, and Ser300-Val308, Glu327-Lys334, and Ile380-Leu387 in the pentamer. The trajectory RMSD of the trimmed structures was analyzed using Hierarchical Clustering Analysis (HCA), employing the Ward variance method. The structures were clustered, and then a dendrogram was produced. The elbow method was applied to ascertain an optimal number of clusters for each system, to ensure representativeness of side chain rotamers.^48^

### Pockets Mapping by the FTMap Server Family

The in silico solvent mapping of protein E was performed using three distinct protocols: (i) a protein mask was applied within the advanced options in the FTMap server^49^ to restrict solvent mapping to regions previously identified as critical for GAG binding—an essential step in dengue virus host cell invasion that promotes clathrin-mediated endocytosis through membrane invagination;^50,51^ (ii) structures were trimmed to the binding site surrounding GAGs, and solvent mapping was carried out as described above; (iii) Atlas (https://acpharis.com/computational-solvent-mapping/), the stand-alone version of FTMap, was used to perform solvent mapping on the whole biological assembly. Additionally, the trimmed structures were submitted to E-FTMap^52^ to identify putative pharmacophore features. The Protein–Ligand Interaction Profiler (PLIP)^53^ was used to calculate and extract interactions between each probe from E-FTMap and the mapped residues within the protein. The intermolecular interactions calculated in this step included hydrogen bonds, hydrophobic contacts, π-stacking, π-cation interactions, salt bridges, water bridges, and halogen bonds. These outputs were compiled with Python 3.11,^54^ and chart generation was performed using Microsoft Excel.

### Hotspots Classification and Analysis

The in-house version of DrugPy plugin,^55^ named XDrugPy, for PyMOL^56^ was used to analyze the groups of consensus sites generated by the protocols described above, supporting the identification of Druggable (Class D) and Borderline druggable (Class B) hotspots, as defined by Kozakov et al. (2015).^57^ Using XDrugPy, residues associated with druggable and borderline hotspots located within 4 Å of the probes within the hotspots were quantified and employed to assess the similarity of the regions surrounding the hotspots. Pairwise similarity (*s*) was evaluated using the Jaccard index between the surrounding residues, and hierarchical clustering was performed based on the corresponding distance metric (*d =* 1 – *s*).

### Sequence Alignment and Conservation Mapping

Sequence alignment of 32 sequences, among different DENV serotypes and other flaviviruses, was performed with the UniProt server (http://www.uniprot.org/), to gauge the conservation of the main residues that make up the described pockets.

For conservation mapping on cryo-EM structure, multiple sequence alignments (MSAs) were performed using UniProt sequence sets to evaluate conservation patterns across flaviviruses and within dengue serotypes. Three MSAs were generated: (i) an interspecies alignment composed of 32 sequences from representative flaviviruses; (ii) an interserotype alignment including 29 sequences from the four DENV serotypes; and (iii) an intraserotype alignment comprising 14 DENV-2 sequences. Conservation scores for each residue were computed in ChimeraX using the built-in sequence conservation tool and subsequently projected onto the trimeric and pentameric assemblies of protein E.

## Results and Discussion

### Structural Dynamics of the Protein E Biological Assemblies

Although the cryo-EM structure of the virion provides unique insights into the organization of the protein E biological assembly, its low resolution makes the positioning of flexible side chains, such as Lys291, rather uncertain. In fact, the Lys291 rotamer observed in the trimeric PDB structure has its positively charged side chain buried toward the interior of the viral particle across all three monomers, which may hinder receptor recognition (Supporting Information Figure S2). To address this limitation, we performed molecular dynamics simulations of both the trimeric and pentameric biological assemblies. Since these models differ from those found in the virus particle (specifically, lacking the other virus particle components, which would interact with the biological assemblies and stabilize the protein complexes), constraints were applied to the protein backbone to keep it close to its original arrangement/folding while allowing the side chains to move freely. As the main goal of this approach is to relax the side chains—a process expected to occur on a time scale between femtoseconds and nanoseconds—each system was simulated for 10 ns.^58,59^

The all-atom RMSD analysis of the trimer suggested that the side chains converged to a stable RMSD value at approximately 6 ns (see Supporting Information Figures S3 and S4). To further characterize side chain conformational changes, we focused on Lys291, a central residue within the assembly, which was previously reported as essential for cell-surface GAG binding in a site-directed mutagenesis assay.^19^ Notably, distinct conformational states were identified for Lys291 throughout the simulation. For chains A and C, RMSD values remained stable throughout the simulation, with the side chain amine group of the lysine residues oriented outward from the viral particle and away from each other, in contrast to the arrangement seen in the cryo-EM original structure. In chain B, however, Lys291 adopted a different rotamer, with its distal amine also oriented outward (Figure 2a and Supporting Information Figure S5). The pentamer simulation displayed a similar profile, with the RMSD values suggesting convergence and indicating side chain rotamer stability at approximately 6 ns. The central residue in the pentameric assembly, Glu383, adopted a conformation distinct from that observed in the cryo-EM structure over the simulation time scale. Prior to the simulation, all Glu383 side chains were oriented toward the interior of the viral particle; however, during the dynamics, the side chains of three subunits reoriented outward, while the remaining two remained inward-facing (Figure 2b and Supporting Information Figure S5).

**Figure 2:**
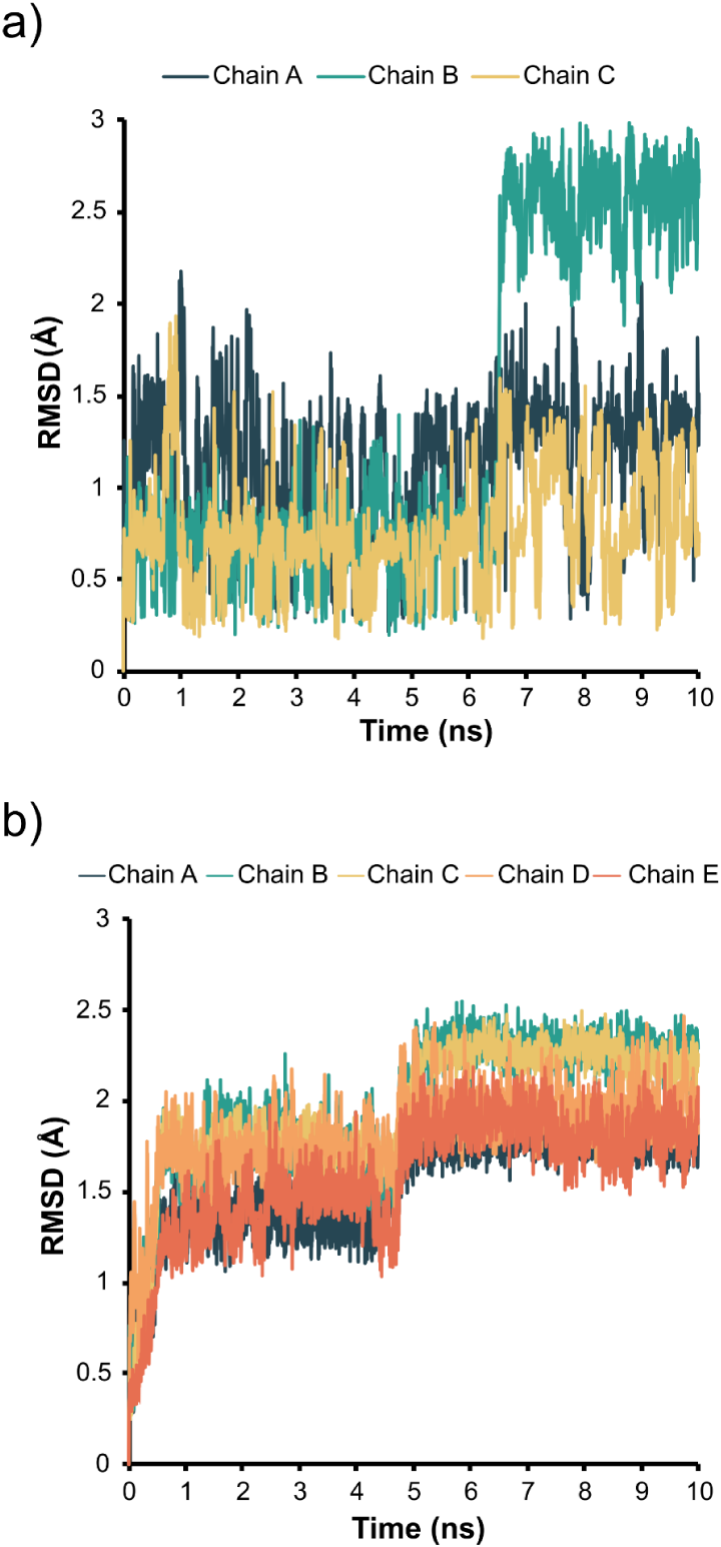
Trimer and pentamer side chain rotamer fluctuation: (a) RMSD values of trimer Lys291 throughout the MD simulations, plotted as a function of the trajectory time; (b) RMSD values of pentamer Glu383 throughout the MD simulations, plotted as a function of the trajectory time.

### Protein E Mapping in the Viral Particle

The *in silico* solvent mapping of the trimer and pentamer using the complete biological assemblies proved frustrating, as consensus sites were detected on both the interior and exterior surfaces of the DENV particle, or the jobs failed altogether due to their large number of atoms. To avoid detecting inaccessible pockets in the interior regions, we designed simplified (trimmed) structures that retain the GAG-binding residues within domain III (DIII)—a region critical to the Dengue virus invasion cycle (Supporting Information, Figure S6). Figure 3 highlights these regions as surface representations on the complete biological assemblies. Solvent mapping was then performed on the trimmed structures for 16 representative side chain trimer conformations and four representative side chain pentamer conformations, as determined by elbow-plot analysis of the hierarchical clustering (HCA) of the trajectory (Supporting Information, Figure S7).

**Figure 3:**
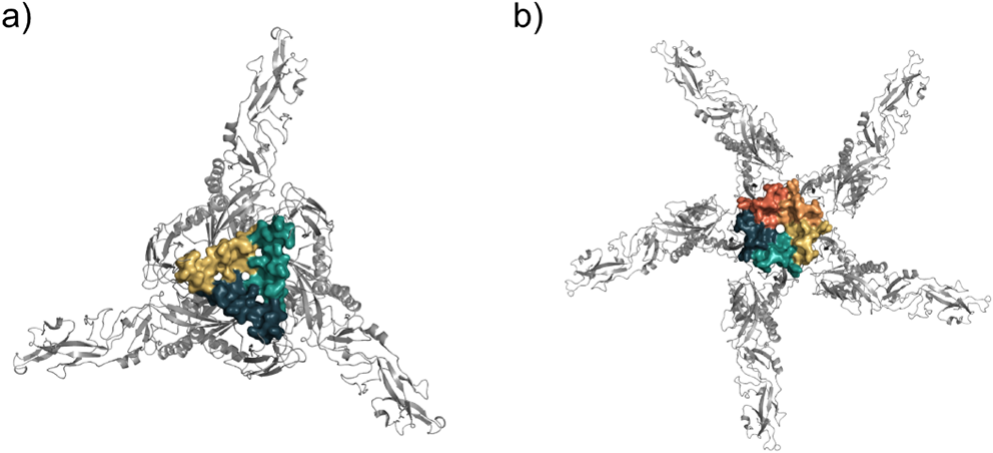
Simplified structure of the representative biological assemblies submitted to in silico solvent mapping. The cartoon representation depicts the complete trimer structure, with the surface highlighting the region analyzed during pocket mapping: (a) trimer; (b) pentamer.

The identified consensus sites, along with their respective locations and compositions, are detailed in Supporting Information Table S2 and Figures S8–S12. Although the mapping effort focused exclusively on the outer surface, some probe clusters were also detected within the interior of the particle. However, only the external consensus sites were retained for subsequent analyses.

Hotspot druggability was assessed using FTMap-derived data from the trimmed representative structures, according to the criteria established by Kozakov et al. (2015).^57^ Hence, based on FTMap-derived data, hotspots (and their corresponding pockets) were classified as *Druggable* (Class D) when they exhibited a high likelihood of binding drug-like compounds with nanomolar affinity. In contrast, *Borderline druggable* hotspots (Class B) were defined as those likely to bind drug-like compounds with at least micromolar affinity. A standardized naming convention was adopted for all hotspots, comprising three elements: the druggability class (*e.g.*, druggable pocket–DP), the identifier of the HCA-derived cluster (*e.g.*, 16th cluster–C16), and the origin of the biological assembly used (*e.g.*, trimer–T), resulting in hotspots such as PD-C16-T (discussed below).

To gain further insight into the nature of these binding pockets, we submitted the trimmed structures to E-FTMap to rationalize potential interaction profiles and support the construction of pharmacophoric models for the respective biological assemblies (Support Information, Table S3). E-FTMap offers enhanced accuracy in predicting intermolecular interaction patterns compared to FTMap.^52^ While the spatial precision of probe positioning may vary depending on interaction type, our primary focus was to identify residues potentially involved in molecular recognition, rather than exact probe placement, particularly in the absence of cocrystallized ligands. All interactions were interpreted from the probe’s perspective, in accordance with the methodology established by the FTMap server family.

To complement these analyses, we employed PLIP to systematically map key interaction types. Among the characterized interactions, hydrogen bond donors (HBDs) and acceptors (HBAs) were further categorized by origin, either in the side chain or the backbone. This distinction is particularly relevant in the context of drug resistance, where mutations frequently alter side chain chemistry while preserving the backbone structure. For instance, Li *et al.* (2018) reported a missense mutation in anaplastic lymphoma kinase that conferred resistance to crizotinib by disrupting a lysine side chain interaction.^60^ Similarly, resistance in enoyl-[acyl-carrier-protein] reductase has been attributed to mutations disrupting side chain contacts.^61^ In the case of protein E, distinguishing between side chain and backbone interactions may be informative for comparative sequence analyses across DENV serotypes and related flaviviruses.

### Identification of Binding Hotspots in the Trimeric Assembly

XDrugPy allowed the identification of one Class D hotspot, containing 18 probes on the main consensus site, and five Class B hotspots, each displaying 14 or 15 probes on the main consensus site (Figure 4a, Support Information Figures S13, S14 and Supplementary Table S2). Analysis of the side chains’ conformational dynamics revealed interchangeable behavior among the identified pockets. For instance, the Class D hotspot DP-C16-T (Figure 4a and b), located near chain A, was also mapped as two distinct Class B hotspots under alternative conformations (BP-C2-T/BP-C3-T and BP-C6-T). These structural variations were primarily associated with rotation of the Glu174 side chain and the nitrogen atom of the ε-amino group in the Lys291 side chain, indicating that, for protein E, conformational changes affecting the interaction profile of probe clusters might significantly impact its druggability (Figure 5b). Minor modifications in the CSs′ center-to-center and maximal distances may also affect hotspot classification.^62^ Nevertheless, such variations could potentially be stabilized through ligand interactions, which were absent in our simulations, thus favoring specific conformational states.

**Figure 4:**
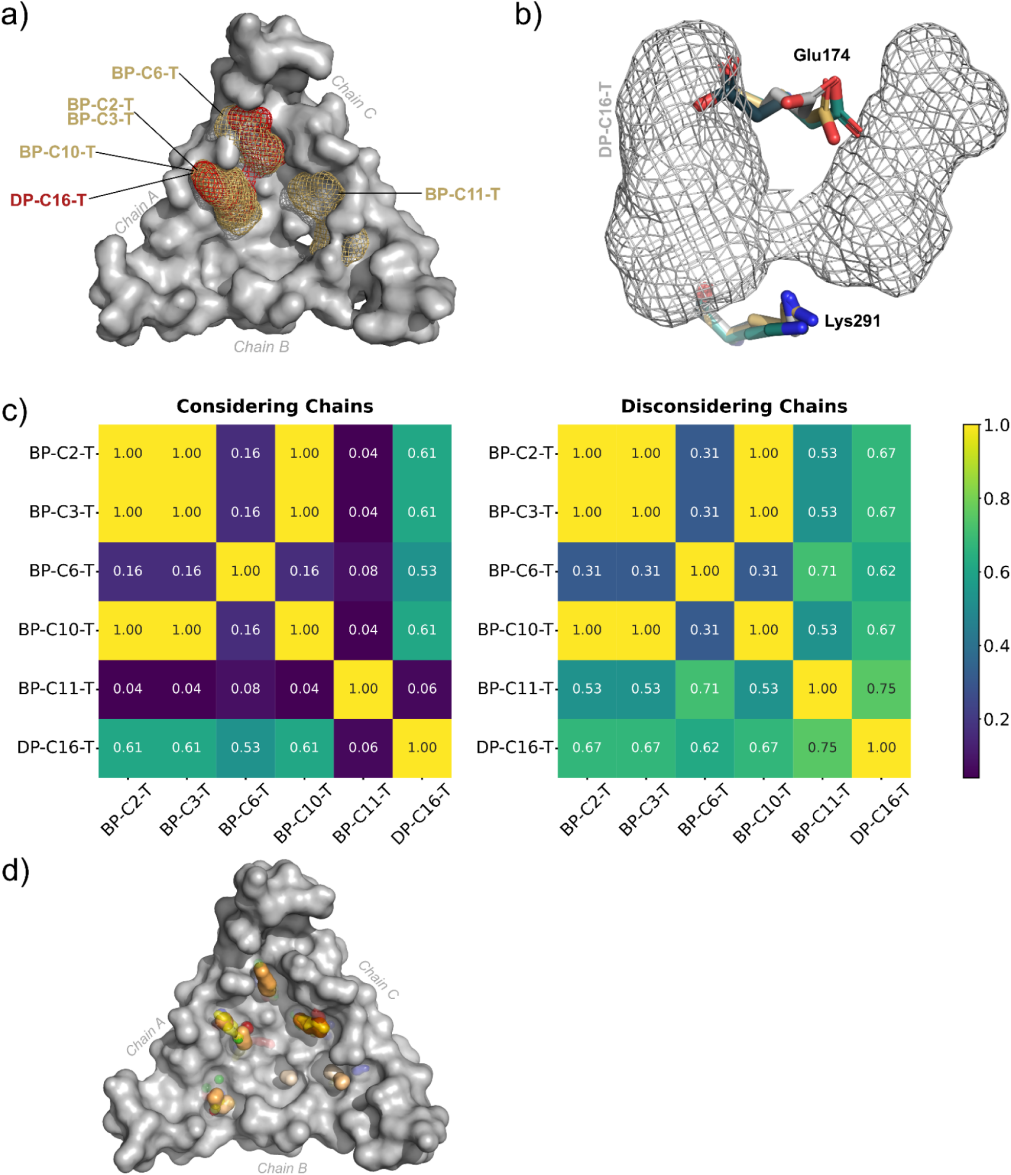
Hotspot analysis in the trimeric biological assembly: (a) positioning of the probes superposition in mesh to represent druggable (red) and borderline druggable (wheat) pockets in the trimer mapped with XDrugPy and FTMap data placed on top of C16-T conformation; (b) C16-T conformation and DP-C16-T pocket (gray) as well the conformational variability of Glu174 and Lys291 among C2 (wheat), C3 (teal), C6 (dark blue); (c) heatmaps displaying the similarity between pockets mapped with XDrugPy, with or without chain differentiation; (d) E-FTMap Atomic Consensus Sites (ACS) for C5 conformation of the trimer assembly.

**Figure 5:**
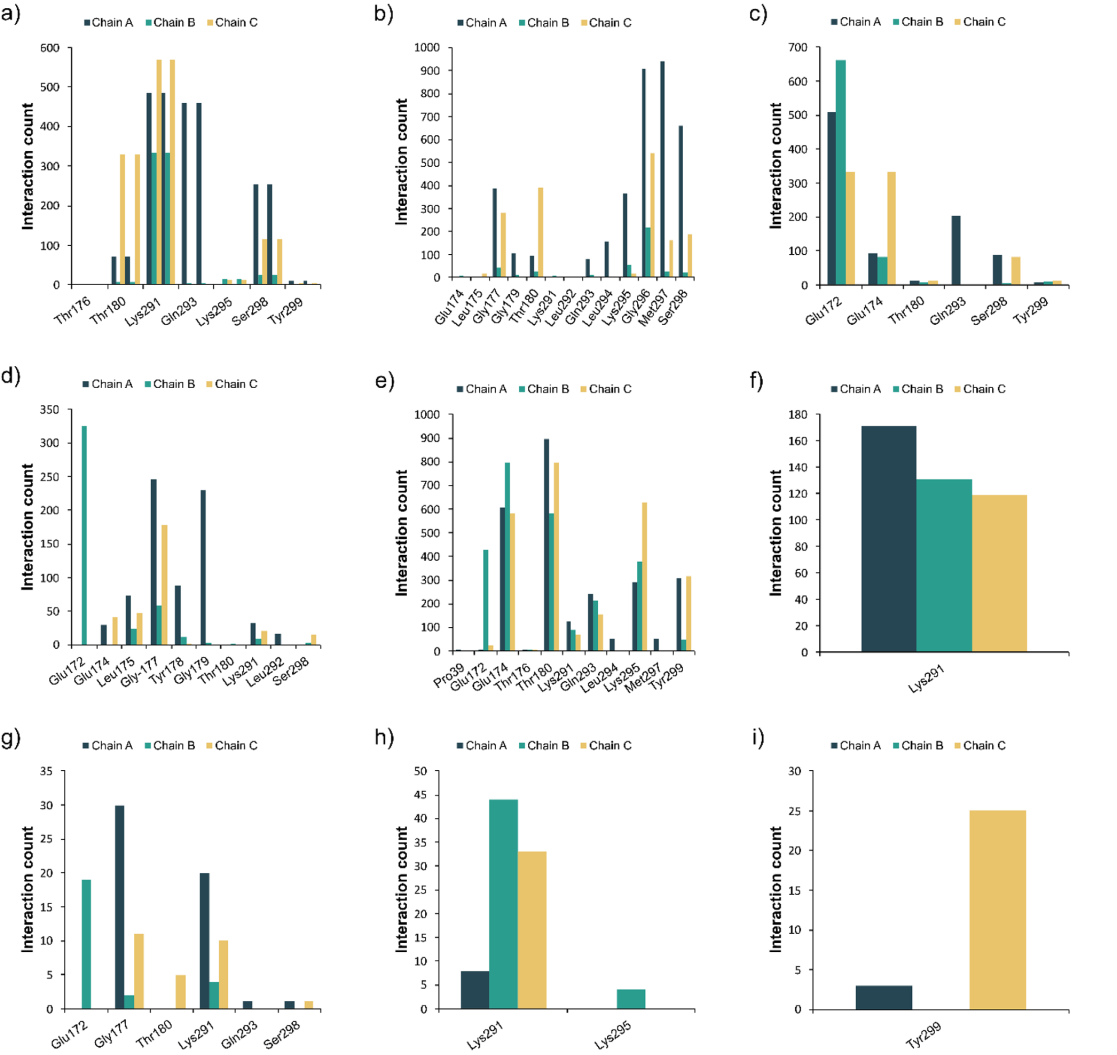
Interaction count from the E-FTMap probes in the trimer mapped structures: (a) Hydrogen bond acceptor (HBA) interactions with the protein side chain; (b) HBA interactions with the protein backbone; (c) Hydrogen bond donor (HBD) interactions with the protein side chain; d) HBA interactions with the protein backbone; (e) hydrophobic interactions; (f) Ionic interaction; (g) halogen bonds; (h) π-cation interactions; (i) π-stacking.

In addition, hotspot BP-C11-T was considered dissimilar to DP-C16-T (0.06 score); however, when the system’s symmetry is considered, the two pockets were closely resembled, displaying a similarity score of 0.75. Also, both pockets combine features observed in BP-C2-T and BP-C6-T (Figure 5c and Support Information Figure S13). Moreover, pockets BP-C2-T, BP-C3-T, and BP-C10-T were located in the same region and displayed identical residue compositions, with similarity scores of 1.00 (Figure 5c and Support Information Figure S14).

A summary of the observed interactions combining the E-FTMap results and PLIP analysis is described in Figure 5. The highest frequencies of hydrogen bond formation were observed at Lys291 (side chain interacting with HBA probes), Gly296, Met297, and Ser298 (backbones interacting with HBA probes), as well as Glu172, which formed interactions with HBD probes through both its side chain and backbone (Figure 4). All these residues faced toward the interior of the previously identified pockets, and as mentioned before, Glu174 and Lys291 could play an important role in the pocket’s druggability.

Hydrophobic interactions were frequently detected at Thr180, Glu174, and Lys295. Notably, Lys291 and Lys295 play a critical role not only due to their capacity to engage in hydrogen bonding, but also in charged interactions, as both residues could form π-cation interactions. Additionally, Lys291 can form ionic contacts. These residues have been previously identified as essential for cell-surface GAG binding.^19^ Given their biological relevance and versatility in mediating diverse noncovalent interactions, they represent promising pharmacophoric regions. Furthermore, residue Thr180 is present in hotspot DP-C16-T and bridging hotspots BP-C2-T and BP-C6-T. Ligand binding to Thr180, along with Lys295, Ser298, Glu174, and Lys291, could therefore contribute to stabilization of a conformation responsible for a druggable pocket (DP-C16-T).

PLIP analysis highlighted the interface between chains A and B as a potentially relevant region, revealing potential interaction sites despite the absence of a well-defined pocket in this area. This observation is further supported by E-FTMap results, which revealed densely packed Active Consensus Sites (ACSs) not only within previously identified pockets but also in a corresponding region on chain B that had not been detected by FTMap (Figure 4d). This probe accumulation can be observed in the C5 conformer of the trimer, which exhibited the highest amount of ACSs on the protein E surface. Together, these findings suggest that this interface may represent a transient binding region. Based on the trimmed biological assembly, a consensus region comprising residues Glu172, Glu174, Tyr178, Thr180, Lys291, and Lys295 was identified, consistently observed across different structures and mapping strategies. Collectively, these findings highlight a structurally and functionally promising region for rational drug design campaigns.

### Identification of Binding Hotspots in the Pentameric Assembly

XDrugPy identified two Class D hotspots, DP-C1-P and DP-C3-P, containing 31 and 28 probes, respectively (Figure 6a, Support Information Figures S13 and S15, and Supplementary Table S2). Additionally, one Class B hotspot, BP-C2-P, was detected, comprising 15 probes. Owing to system symmetry, a behavior similar to that observed in the trimeric assembly was noted. Specifically, the pockets occupied the interfacial space between two protein E monomers, depending on their conformational state. DP-C1-P is located between chains A and E, DP-C3-P is positioned between chains D and E, and BP-C2-P lies between chains B and C. Despite being located in different chains, when considering only the residues for similarity analyses, the pockets display similarity scores of 0.75 or higher (Figure 6b). This is a relevant consideration in homomultimeric systems, where symmetric assemblies should yield equivalent surface areas across different portions of the structure. Hence, the position of the druggable pockets is not specific to a given chain. Moreover, from E-FTMap ACSs, it is possible to observe sites almost evenly spaced between the chains (Figure 6c).

**Figure 6:**
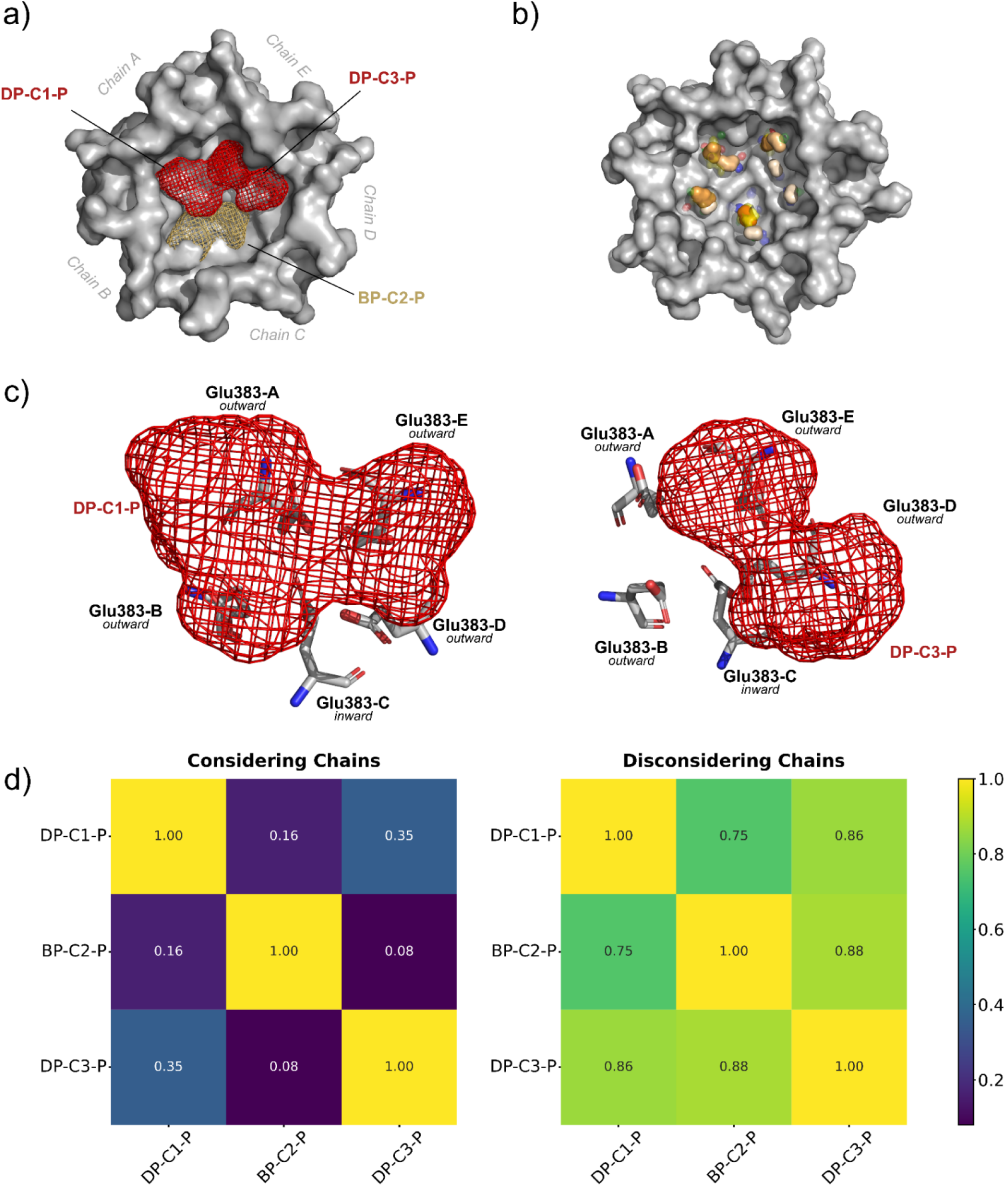
Hotspot analysis in the pentameric biological assembly: (a) positioning of the probes superposition in mesh to represent druggable (red) and borderline druggable (wheat) pockets in the pentamer as calculated by XDrugPy and FTMap data placed on top of C1-P conformation; (b) E-FTMap Atomic Consensus Sites (ACS) for C2-P positioning on the pentameric assembly; (c) Glu383 positioning within DP-C1-P and DP-C3-P; (d) heatmap displaying the similarity between pockets mapped with XDrugPy, with or without chain differentiation.

A summary of the interactions observed in the pentameric assembly is presented in Figure 7. Hydrogen bonds were detected at multiple residues, with Glu383—the central residue of the pentamer—being the most frequently involved. Positioned at the base of the identified pockets, the three outward-facing Glu383 side chains were included in pocket DP-C1-P. In contrast, in DP-C3-P and BP-C2-P, only two Glu383 residues from distinct chains contributed to the pocket shape (Figure 6d). This behavior contrasts with that observed in the trimeric assembly, where the central pocket region is predominantly positively charged, while in the pentamer it is negatively charged due to the presence of Glu383. Furthermore, probes interacting with this residue formed hydrogen bonds with both the backbone (as hydrogen bond acceptors) and the side chain (as hydrogen bond donors). In contrast, interactions involving Gln386 primarily engaged its side chain, acting as a hydrogen bond acceptor.

**Figure 7:**
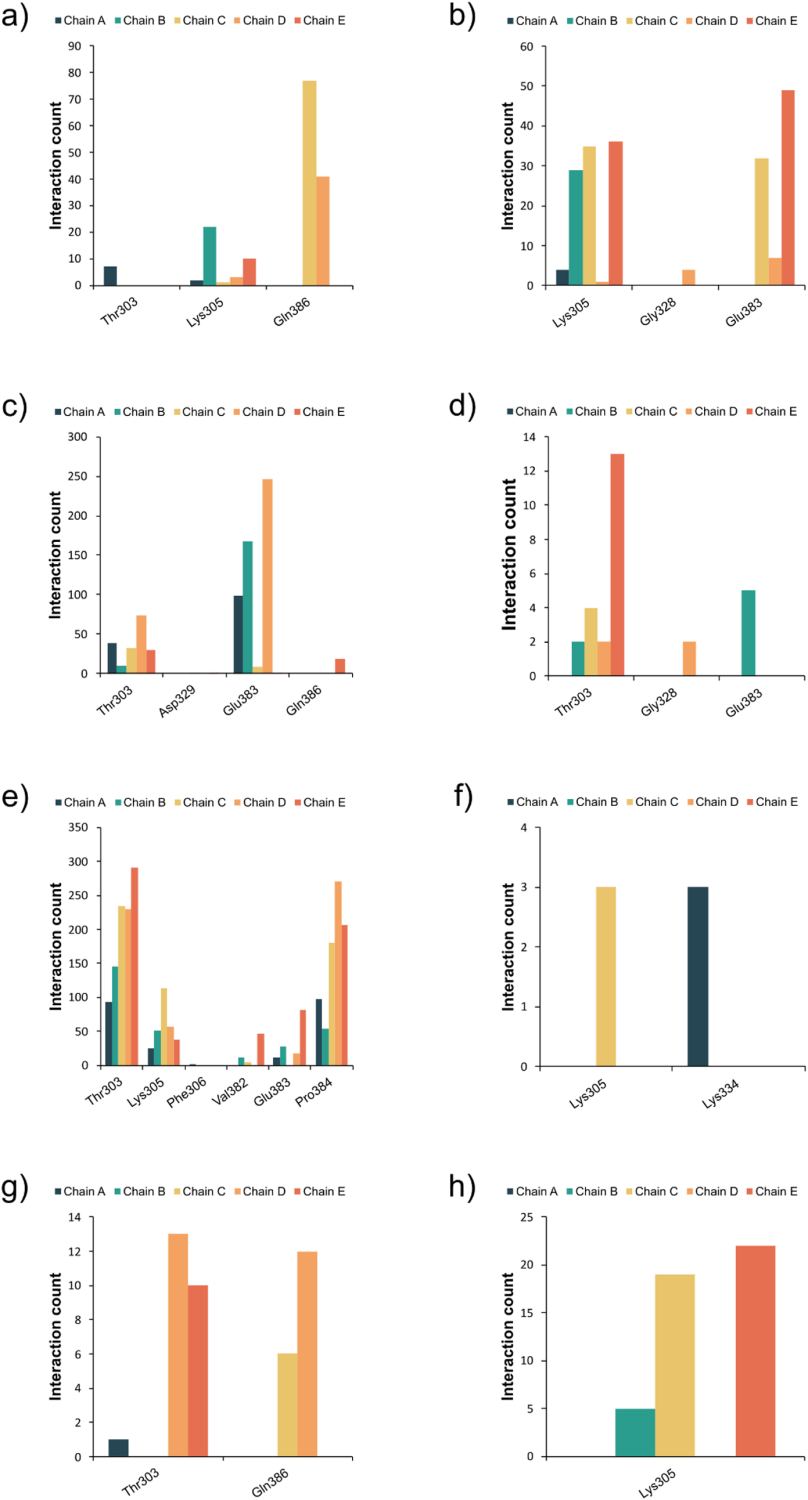
Interaction count from the E-FTMap probes in the pentamer mapped structures: (a) Hydrogen bond acceptor (HBA) interactions with the protein side chain; (b) HBA with the protein backbone; (c) Hydrogen bond donor (HBD) interactions with the protein side chain; (d) HBD interactions with the protein backbone; (e) hydrophobic interactions; (f) Ionic interaction; (g) halogen bonds; (h) π-cation interactions.

Thr303 emerged as a prominent hydrophobic contact, with the potential to engage in hydrogen bond donor (HBD) interactions through both its backbone and side chain. Lys305 also contributed significantly, participating in ionic and π-cation interactions. Another notable residue was Pro384, a residue recognized as important to the viral entrance process,^63^ which formed consistent hydrophobic interactions. Interestingly, each pocket contains two Pro384 from distinct chains. Together, Thr303 and Lys305, and Pro384 form a compact hydrophobic cluster that also engages in hydrogen bonding, underscoring the relevance of this surface patch as a promising site for structure-based drug design (Figure 6e).

### Pharmacophoric Features of the Biological Assemblies

The mapped pockets were located at interdomain interfaces that are uniquely formed on the viral particle surface and are absent in isolated monomeric or dimeric structures. This structural context justifies their selection as primary targets for mapping, as it better reflects the native assembly state encountered during infection and offers access to previously uncharacterized binding sites. We observed that only 40% of the trimeric representative structures displayed druggable or borderline druggable hotspots, whereas this proportion reached 75% in the pentamer. This difference may suggest a trend toward lower druggability in the trimeric assembly under the conditions analyzed. We should exercise caution in concluding that this difference in druggability is significant, as no reported major folding changes have been observed due to the presence of ligands in trimeric and pentameric assemblies in the literature. Therefore, simulations of apo and holo structures may be required to support this comparison and its respective conclusions.

Based on the physicochemical nature and spatial arrangement of the interacting residues, we propose distinct pharmacophoric features for the trimeric and pentameric assemblies. For the trimer, a representative feature set could exploit the side chain of Glu172 acting as a hydrogen bond donor, Thr180 as a hydrophobic contact, the side chain of Lys291 as a hydrogen bond acceptor, and Lys295 as a hydrophobic contact (Figure 8a). In contrast, in the pentameric model, Thr303 and Pro384 may be explored as hydrophobic contacts, Glu383 as a hydrogen bond acceptor via backbone interactions, and a hydrogen bond donor via the side chain. Additionally, Lys305 due to its capability of interacting by ionic and π-cation interactions, and hydrogen bond acceptor via the backbone (Figure 8b). These residues delineate chemically diverse and spatially defined features that can guide future structure-based drug design and virtual screening efforts.

**Figure 8:**
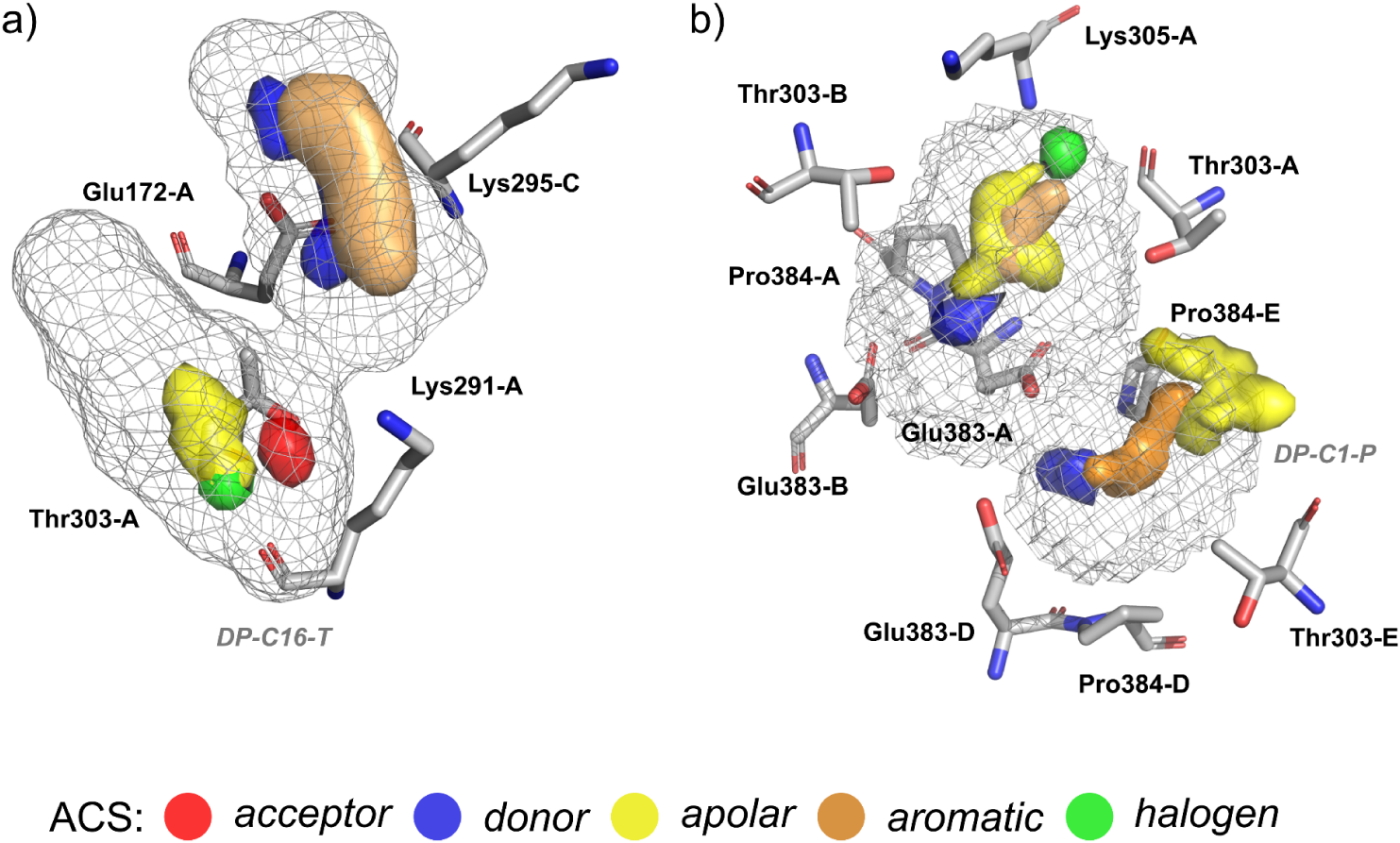
Proposed pharmacophoric features based on druggable pockets for the biological assemblies: (a) trimeric conformation corresponding to pocket DP-C16-T; (b) pentameric conformation corresponding to pocket DP-C1-P. Key interacting residues, pocket mesh, and Atomic Consensus Sites (ACS) surfaces are shown, illustrating the chemical environment around it.

### Sequence Conservation across Flaviviruses and Its Implications for Pocket Targeting

Sequence alignment shows that at the trimeric pocket, residues Lys291 and Lys295 are highly conserved, as are Glu172 and Thr180, to a lesser extent (Support Information Figure S16). The lysine residues are present in all aligned sequences (except for Lys291 in Tembusu virus and yellow fever virus), corroborating the aforementioned mutagenesis studies.^19^ For the pentameric pocket residues, alignment shows that, for the selected sequences, Thr303 has high conservation while Glu383 and Pro384 are less conserved (Support Information Figure S17).

To assess the evolutionary relevance of the pockets identified in the trimeric and pentameric assemblies, the residue conservation was evaluated across multiple taxonomic levels. When conservation scores from a 32-sequence flavivirus alignment were projected onto the oligomeric assemblies, several of the proposed pharmacophoric residues, including Lys291, Lys295, and Glu172 (trimer), displayed moderate to high conservation. This trend was reinforced in the interserotype alignment (29 DENV sequences) and became even more pronounced in the intraserotype alignment (14 DENV-2 sequences), indicating that the structural and chemical features of these pockets are maintained across circulating viral isolates.

Visualization of the trimeric and pentameric assemblies colored by conservation score (Supporting Information Figure S18) reveals that conserved residues cluster in regions implicated in GAG engagement and receptor-associated interactions. The consistency of these conservation patterns across alignments suggests that ligands designed to target these pockets may retain activity across DENV serotypes and, in some cases, across related flaviviruses. Conversely, pockets with lower conservation, particularly in the pentameric interface, may offer opportunities for serotype-selective inhibitor design. These structure-guided insights support the biological significance and therapeutic potential of the pockets identified in this study.

The mature dengue virion displays a complex surface topology defined by the icosahedral arrangement of 180 envelope proteins. The trimeric and pentameric assemblies surrounding the 3–fold and 5–fold symmetry axes constitute functionally critical host-interaction surfaces. These oligomeric interfaces expose residues essential for attachment to GAGs, including heparan sulfate; mediate initial contact with host receptors; and correspond to neutralizing antibody epitopes. Our structural analysis demonstrates that these regions are not only biologically meaningful but could also be conserved across dengue and related flaviviruses (along the 3–fold symmetry axes), as reflected in the superposition of multiple cryo-EM assemblies (Supporting Information Figure S18).

A key outcome of our analysis is the clear distinction between conserved residues in trimeric pockets and the more variable environment of the pentameric pockets. This dichotomy implies that small molecules targeting the trimeric interface may provide broader antiviral coverage, while pentameric interface inhibitors could confer serotype or strain selectivity. The pharmacophoric elements identified in both assemblies provide a foundation for future structure-based virtual screening and fragment discovery.

## Conclusions

In this work, we used a cryo-EM structure of the mature dengue virion to investigate protein E in its native trimeric and pentameric assemblies, revealing binding sites that are absent in isolated dimers. Studying the protein directly in its biological context allowed us to examine surface regions that are functionally relevant for host-cell attachment, including residues involved in interactions with glycosaminoglycans, as well as epitopes recognized by neutralizing antibodies. Structural comparison of flavivirus assemblies shows that the trimeric interface is highly conserved at the backbone level across dengue and related viruses, underscoring its biological relevance as a potential antiviral target. The pentameric interface, while still exhibiting a conserved geometric scaffold, shows greater residue-level variability, suggesting that pockets in this region may be more amenable to serotype- or strain-selective modulation.

Because cryo-EM models typically contain uncertainties in side chain positioning, especially in flexible or charged residues, we refined the assemblies using restrained molecular dynamics simulations to improve local geometry while preserving the experimentally supported oligomeric architecture. This refinement enabled reliable identification of druggable and borderline-druggable pockets in both assemblies. Key residues forming these pockets, such as Lys291, Lys295 and Pro384, have previously been implicated in receptor engagement or viral entry, reinforcing the biological consistency of the mapped hotspots. By combining cryo-EM assemblies, side chain refinement, and fragment-based solvent mapping, we describe pharmacophoric features that characterize each pocket and can guide virtual screening, ligand design, and repurposing strategies. The strong evolutionary conservation of trimeric-pocket residues suggests potential for broad-spectrum antiviral development, while the more variable pentameric-pocket residues may support selective inhibition of specific serotypes or circulating strains. Our findings reveal previously unexplored, biologically meaningful pockets on the dengue virion surface and lay the groundwork for structure-guided development of protein E–targeted antiviral compounds.

It is important to mention that this study focuses on static oligomeric assemblies and evaluates only local side chain flexibility accessible through short, backbone-restrained simulations. Large-scale conformational transitions of protein E, such as domain rearrangements or temperature-induced surface reorganization, were not investigated here and represent important avenues for future work. The lack of ligand-bound cryo-EM structures also limits direct comparison between predicted hotspots and experimentally validated binding modes. Future studies using enhanced sampling simulations, longer unrestrained trajectories, or biophysical validation of entry inhibitors will help extend and refine the structural framework established here.

## Supplementary Material



## Data Availability

The data associated with this study are available in a Zenodo repository (10.5281/zenodo.15851419), organized as follows: a folder named *MD*, which contains the trajectories for both the trimer and pentamer, and a folder named *Mapping*, which includes clustering data, representative structures, and raw data from FTMap and E-FTMap.

## Notes

Details on the results, along with analyses presented through figures and tables (PDF)

## Abbreviations

ACS: atomic consensus site
B: borderline
cryo-EM: cryogenic electron microscopy
D: druggable
DENV: dengue virus
CYD-TDV: Dengvaxia
D: distance
DI: domain I
DII: domain II
DIII: domain III: envelope protein
GAG: glycosaminoglycan
HCA: hierarchical clustering analysis
HBA: hydrogen bond acceptor
HBD: hydrogen bond donor
MD: molecular dynamics
PME: particle mesh Ewald
PDB: Protein Data Bank
PLIP: Protein–Ligand Interaction Profiler
RMSD: root-mean-square deviation
S: similarity
VMD: Visual Molecular Dynamics
