# Supplementary material for: Reading between the Chains: Surface Mapping and Druggable Pockets on the Biological Assemblies of DENV-2’s Protein E
Source: ACS Omega. 2026 Jan 26;11(5):7518–31. doi: 10.1021/acsomega.5c08709 (PMC12902848; doi:10.1021/acsomega.5c08709)
Supplement: Supplementary file 1 [file ao5c08709_si_001.pdf]

Support Information

Reading Between the Chains: Surface Mapping and Druggable Pockets on the Biological Assemblies of DENV-2's Protein E

Pedro T. T. F. Leite,[1#] Philipe O. Fernandes,[1#] Pedro S. Lacerda,[2#] Marcelo A. Chagas,[3] Marcelo S. Castilho,[2] Willian R. Rocha,[4] Adolfo H. Moraes,[4, 5*] Vinícius G. Maltarollo[1*]

[1] Departamento de Produtos Farmacêuticos, Faculdade de Farmácia, Universidade Federal de Minas Gerais (UFMG), Av. Antônio Carlos, 6627, Belo Horizonte, MG, 31270-901, Brasil
[2] Programa de Pós-Graduação em Farmácia, Faculdade de Farmácia, Universidade Federal da Bahia (UFBA), R. Barão de Jeremoabo 14, Ondina, Salvador, BA, 40170-115, Brasil
[3] Departamento de Ciências Exatas, Universidade do Estado de Minas Gerais (UEMG), Av. Brasília, 1304, João Monlevade, MG, 35930-314, Brasil
[4] Departamento de Química, Instituto de Ciências Exatas, Universidade Federal de Minas Gerais (UFMG), Av. Antônio Carlos, 6627, Belo Horizonte, MG, 31270-901, Brasil
[5] Laboratório de Ressonância Magnética Nuclear (LAREMAR), Universidade Federal de Minas Gerais (UFMG), Av. Antônio Carlos, 6627, Belo Horizonte, MG, 31270-901, Brasil
**Email: viniciusmaltarollo@gmail.com, maltarollo@ufmg.br*
[#] P.T.T.F.L., P.O.F., and P.S.L equally contributed to this work.

Support Information Figure S1. Superimposition of eight cryo-EM backbone models from dengue virus (DENV) serotypes and Zika virus (ZIKV) (PDB: 8Y3G, 8Y3J, 3J6S, 5IRE, 5IZ7, 4CBF, 4CCT, 6CO8), selected for their biological assemblies analogous to PDB 3J27. (A) Trimeric (three-fold) axis. (B) Pentameric (five-fold) axis.

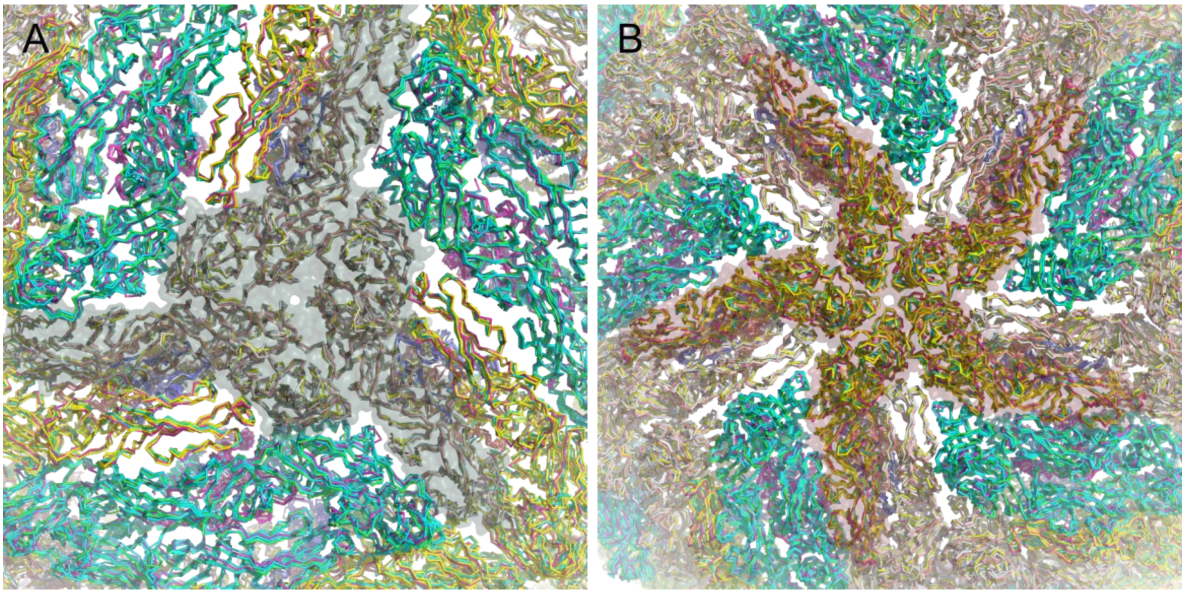

Support Information Figure S2. Steric hindrance of Lys291 in the trimer structure on the viral particle surface: The lysine residues are positioned 6.5 Å apart and oriented toward the interior of the viral particle (PDB ID: 3J27). Given their crucial role in viral functionality, they were expected to face the exterior of the viral particle.

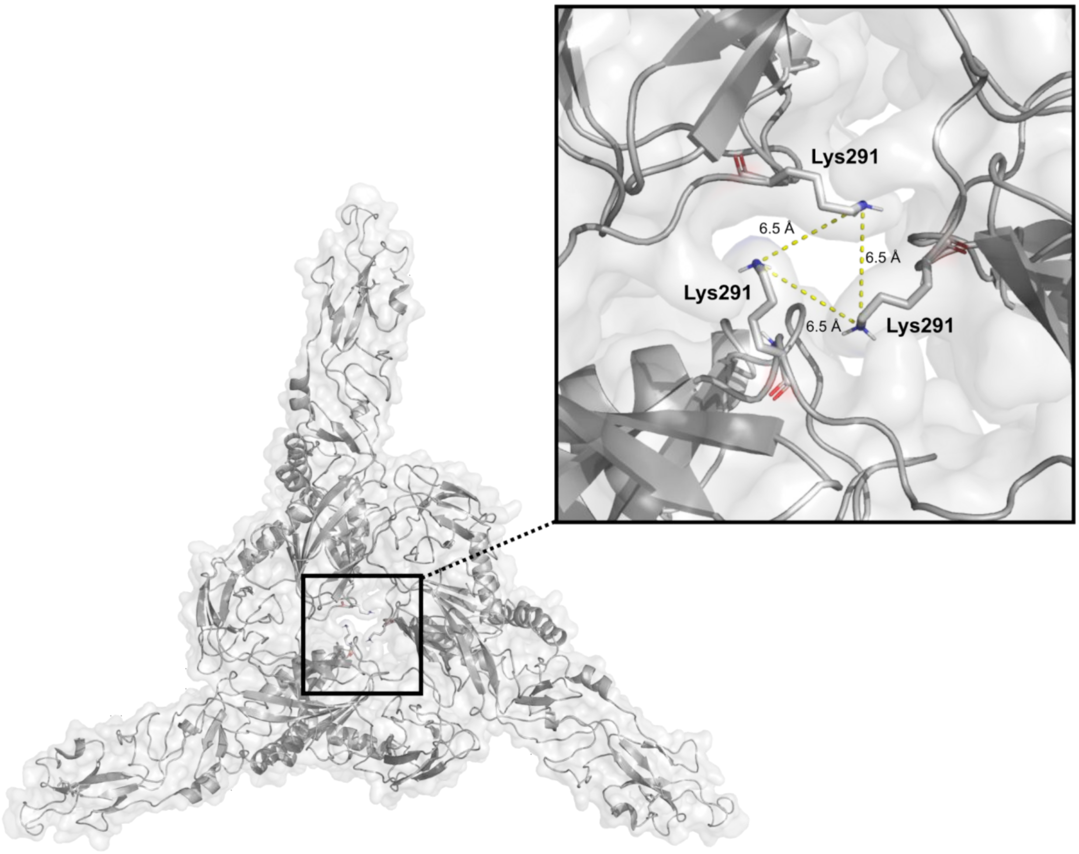

Support Information Figure S3. Side chain rotamer fluctuation via all-atom RMSD analysis on the viral particle surface: The RMSD values from molecular dynamics simulations are presented as a function of the trajectory, split by chain. a) full structure of the trimer; b) trimmed region of the trimer, including Pro39, Thr171–Val181, Lys291–Tyr299, Ser331–Lys334, and Asn355–Thr359 residues; c) full structure of the pentamer; d) trimmed region of the pentamer, including Ser300–Val308, Glu327–Lys334, and Ile380–Leu387 residues.

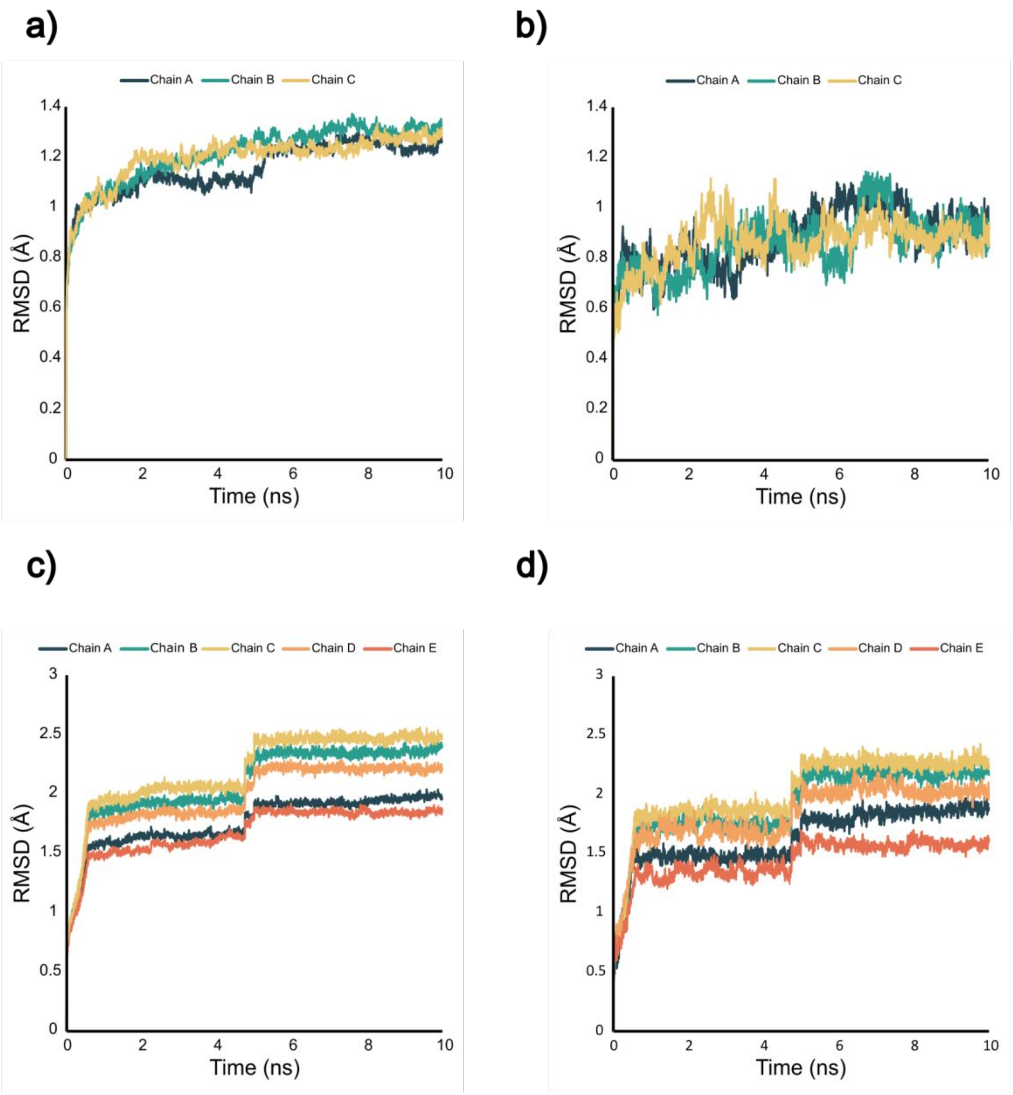

Support Information Figure S4. Side chain RMSF analysis of protein E residues: the RMSF values for each residue are presented, split by chains and averaged. a) trimer side chain RMSF values split by chain; b) trimer average side chain RMSF values; c) pentamer side chain RMSF values split by chain; d) pentamer average RMSF values.

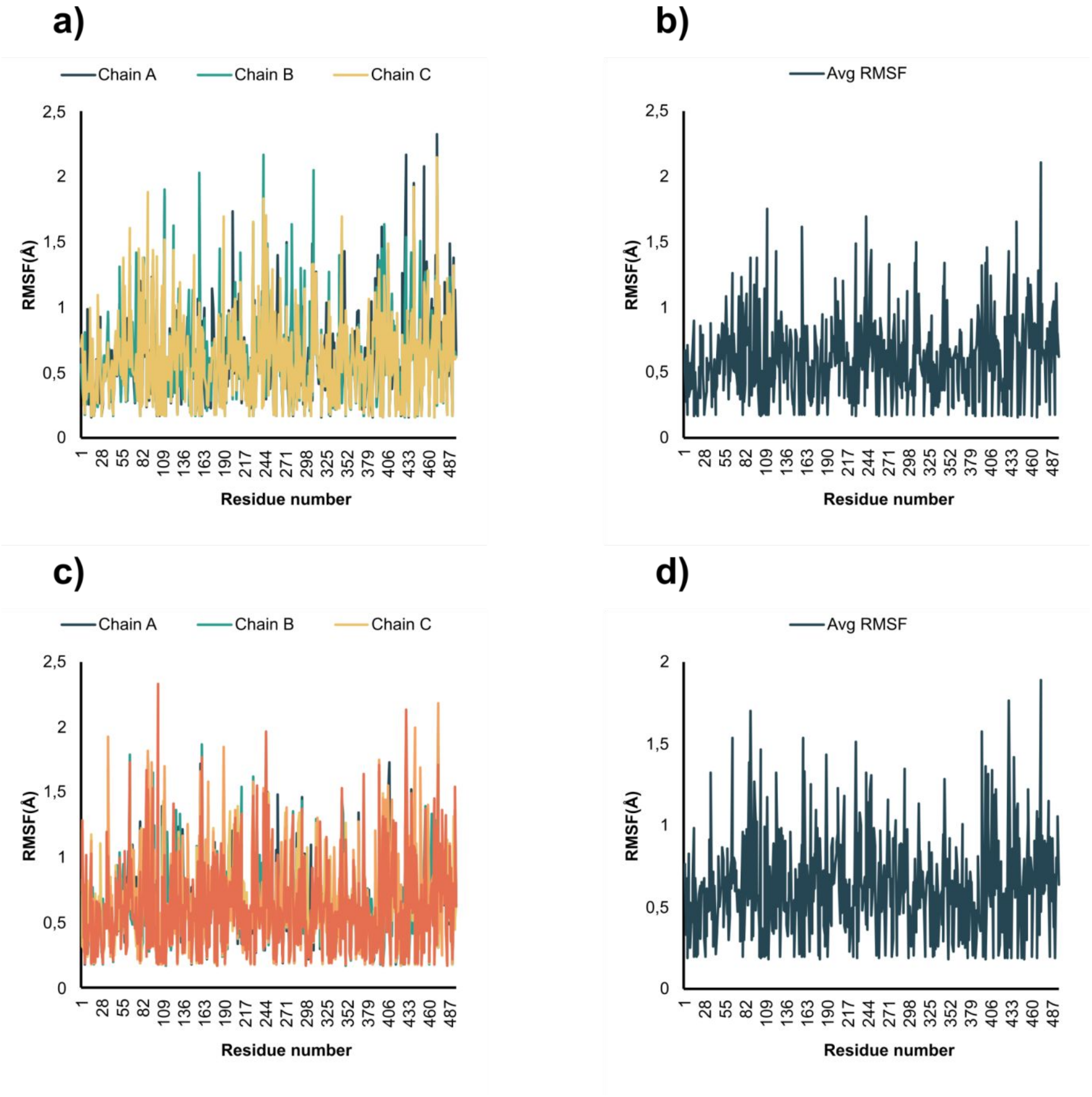

Support Information Figure S5. Conformational variability observed from the Molecular Dynamics trajectory: a) licorice representation of Lys291 in the representative structures extracted from the trimer trajectory at 2.5 ns (yellow), 4.9 ns (cyan), 7.0 ns (green), and 8.5 ns (pink) in comparison to the Cryo-EM structure shown in gray; b) licorice representation of Glu383 in the representative structures extracted from the pentamer trajectory at 0.2 ns (cyan), 0.5 ns (yellow), 2.6 ns (pink), and 9.2 ns (green) in comparison to the Cryo-EM structure shown in gray.

a) b)

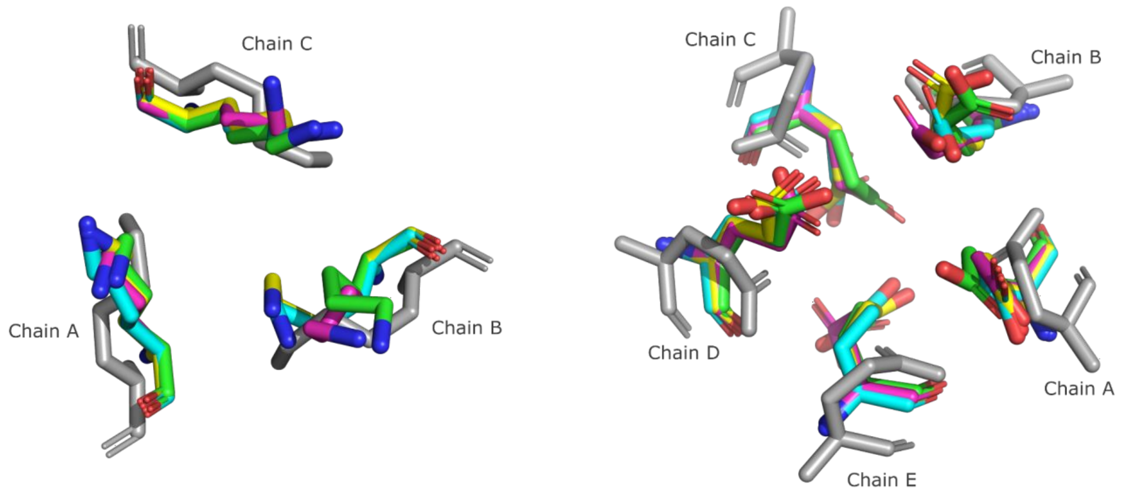

Support Information Figure S6. Mapping of the biological assemblies revealed multiple pockets located inside the virion, which are inaccessible in the mature dengue particle. These internal cavities arise from the transmembrane region, which is positioned on the inner surface of the viral envelope and is responsible for anchoring the E protein to the lipid membrane (PDB ID: 3J27). During preliminary mapping attempts performed directly on the full biological assembly, FTMap probes preferentially filled these internal, membrane-facing cavities, as the enclosed environment provides a more favorable steric environment for the probes. However, these pockets do not represent ligand-accessible sites on the mature virion, because they face the interior of the viral particle. Therefore, only pockets located on the external surface of the trimeric and pentameric assemblies were considered relevant for antiviral design.

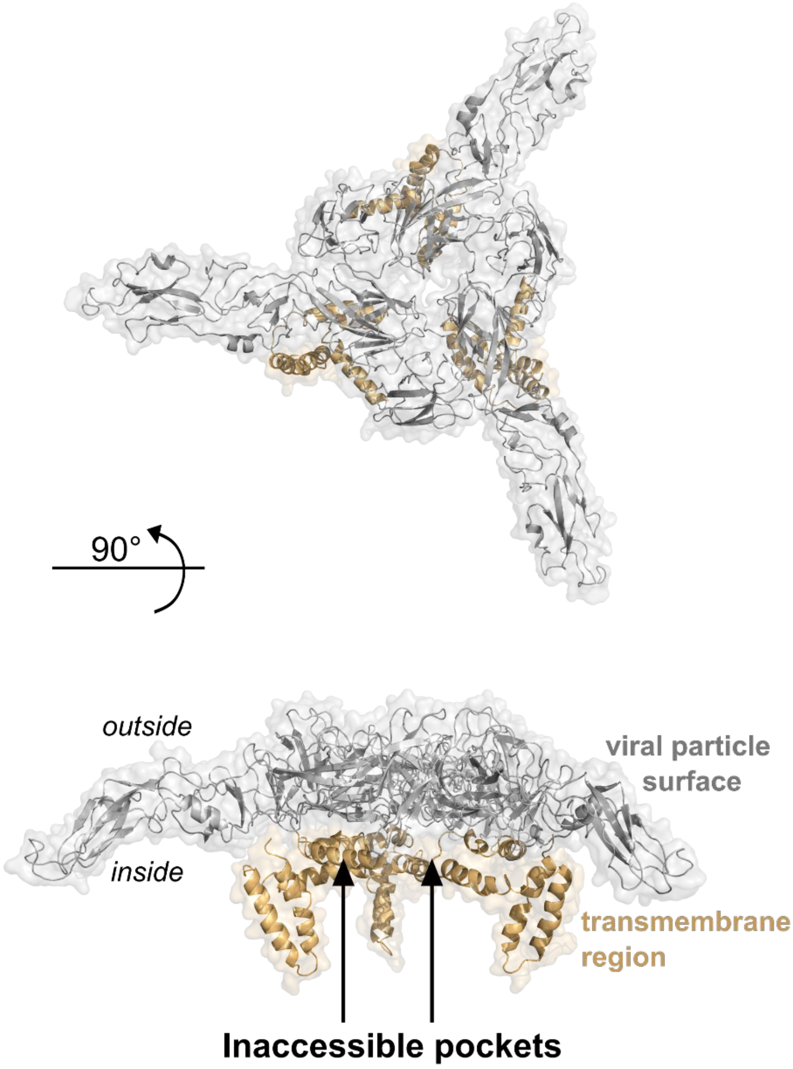

Support Information Figure S7. Molecular dynamics structural clustering using Hierarchical Cluster Analysis (HCA): a) elbow plot illustrating the clustering of the trimer structures; b) elbow plot illustrating the clustering of the pentamer structure; c) HCA dendrogram of the pentamer structures; d) HCA dendrogram of the trimer structures. Five clusters were selected from the molecular dynamics (MD) trajectory, and sixteen from the pentamer MD trajectory.

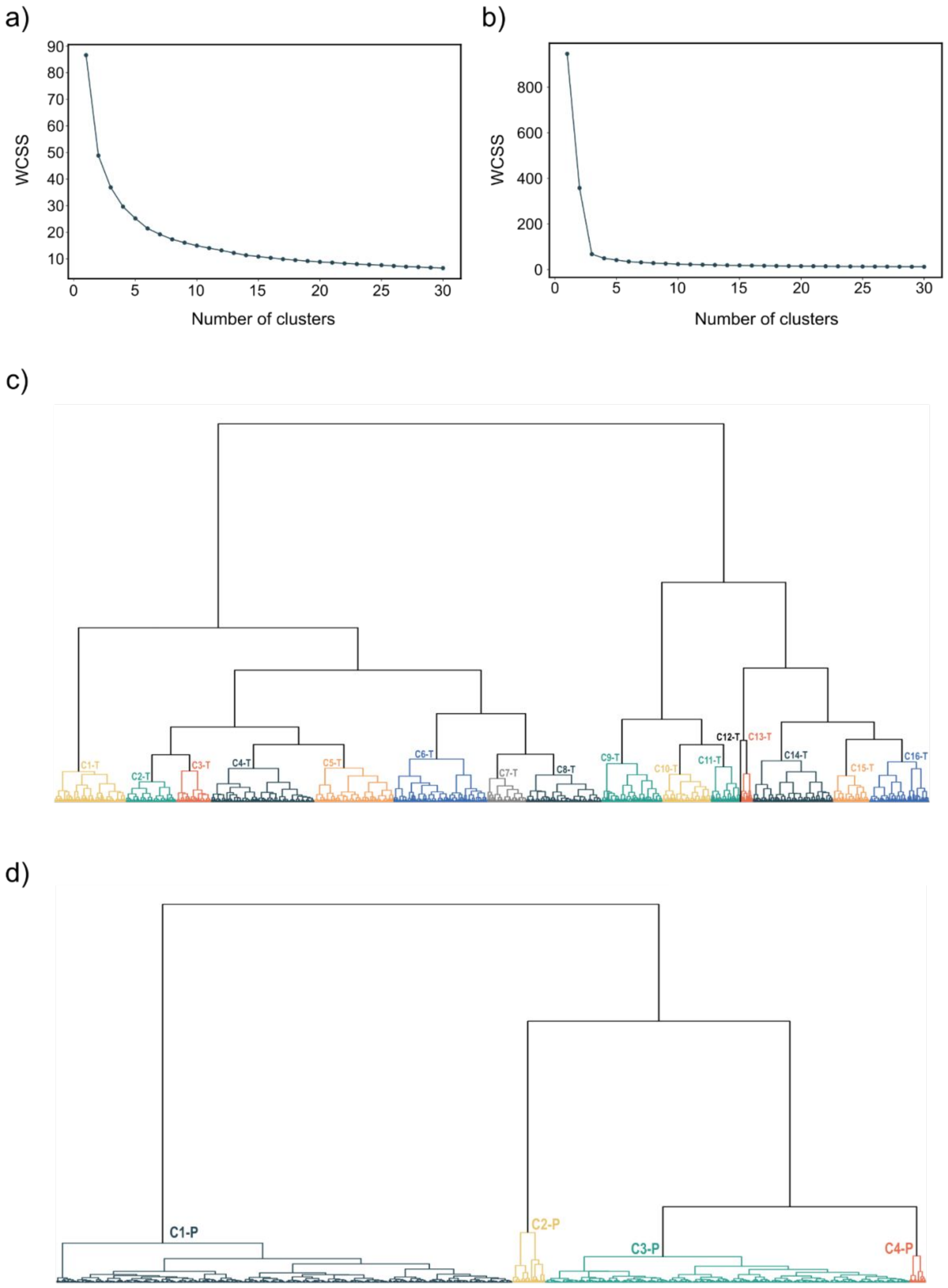

Support Information Figure S8. Binding hotspots identified by FTMap and E-FTMap in the trimer structure for clusters **C1-T** to **C4-T**, shown from both perspectives of the DENV viral particle. The external surface corresponds to the outer side of the viral particle, while the internal surface represents its interior.

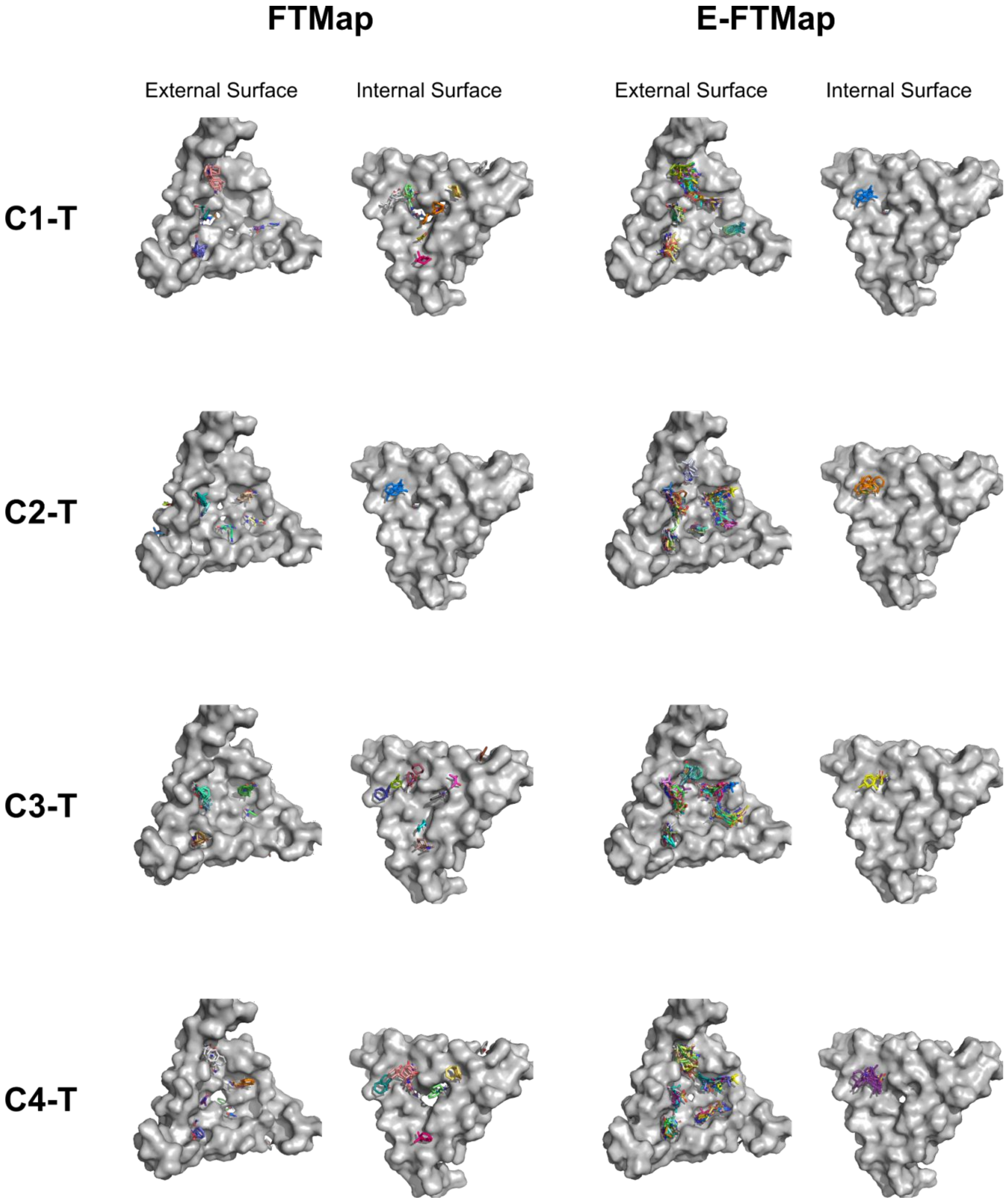

Support Information Figure S9 Binding hotspots identified by FTMap and E-FTMap in the trimer structure for clusters **C5-T** to **C8-T**, shown from both perspectives of the DENV viral particle. The external surface corresponds to the outer side of the viral particle, while the internal surface represents its interior.

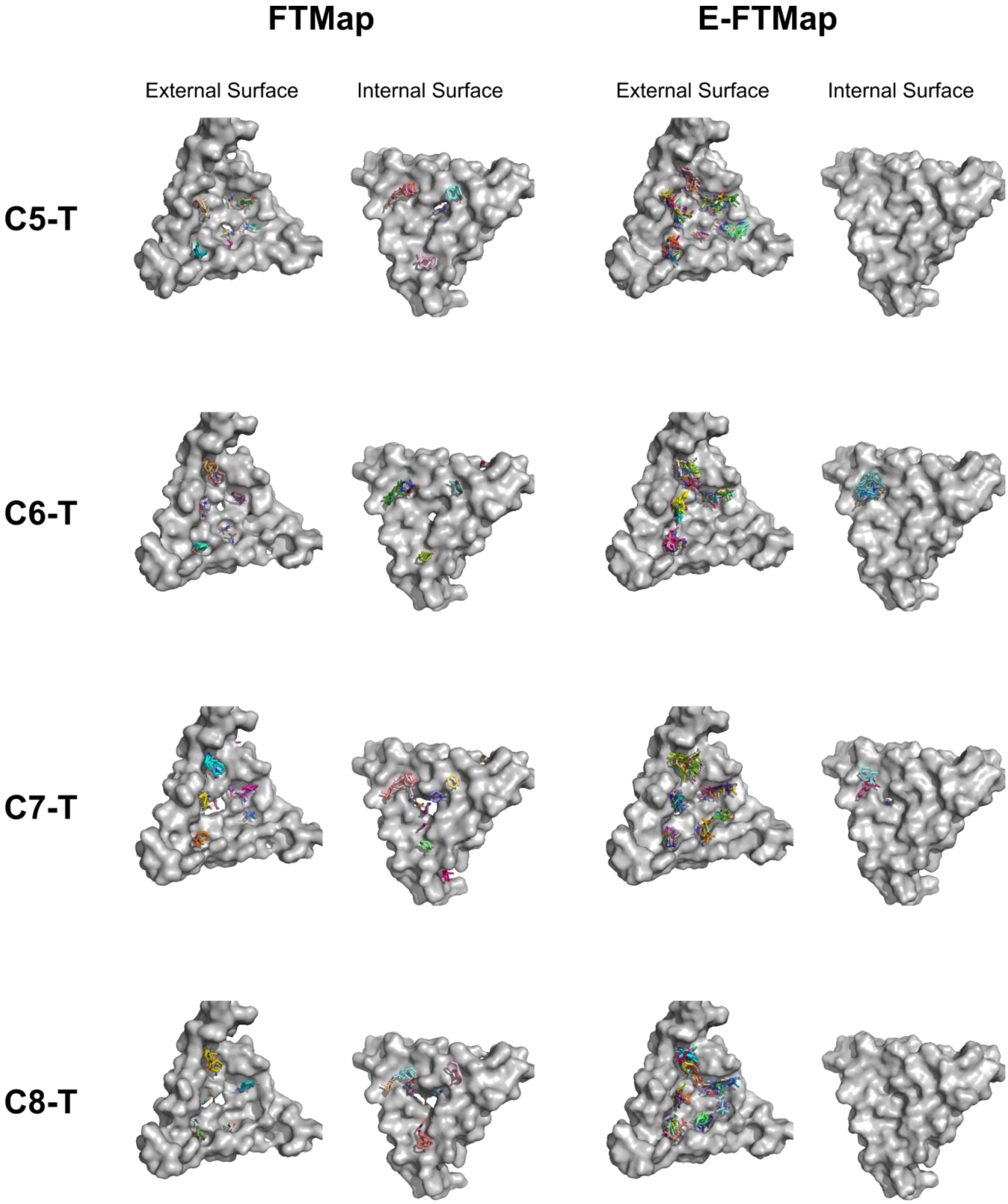

Support Information Figure S10. Binding hotspots identified by FTMap and E-FTMap in the trimer structure for clusters **C9-T** to **C12-T**, shown from both perspectives of the DENV viral particle. The external surface corresponds to the outer side of the viral particle, while the internal surface represents its interior.

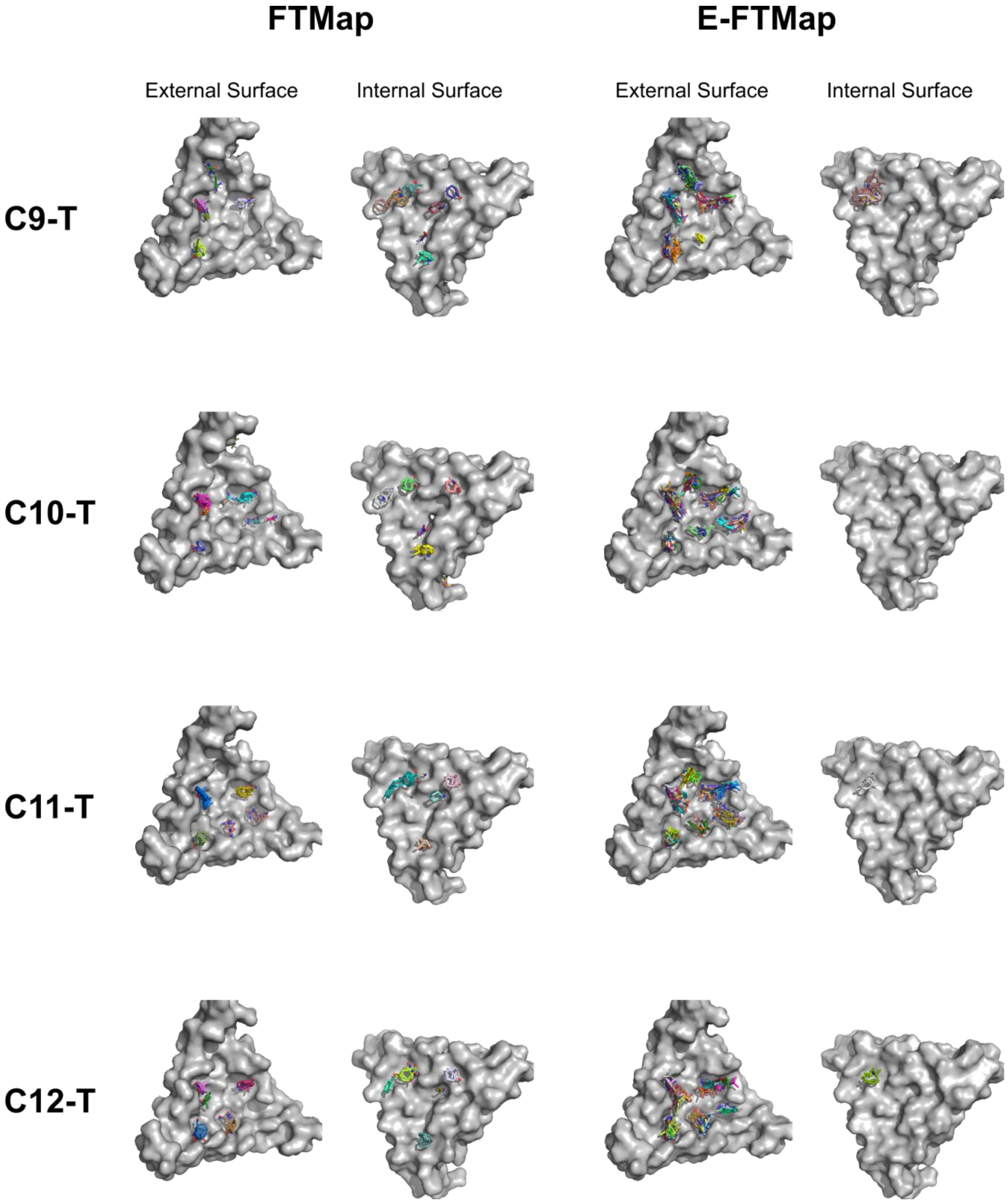

Support Information Figure S11. Binding hotspots identified by FTMap and E-FTMap in the trimer structure for clusters **C13-T to C16-T**, shown from both perspectives of the DENV viral particle. The external surface corresponds to the outer side of the viral particle, while the internal surface represents its interior.

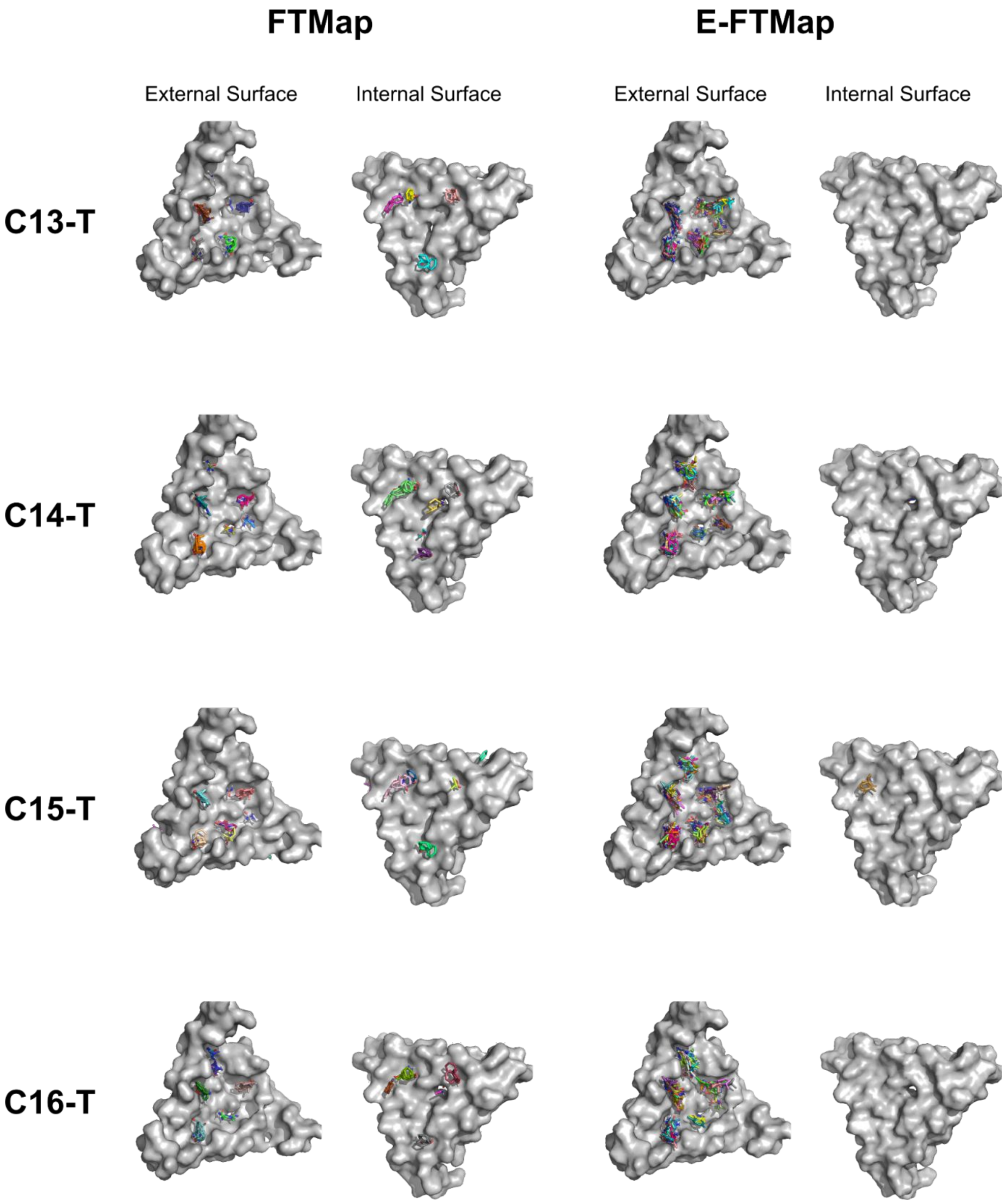

Support Information Figure S12. Binding hotspots identified by FTMap and E-FTMap in the pentamer structure for all clusters, shown from both perspectives of the DENV viral particle. The external surface corresponds to the outer side of the viral particle, while the internal surface represents its interior.

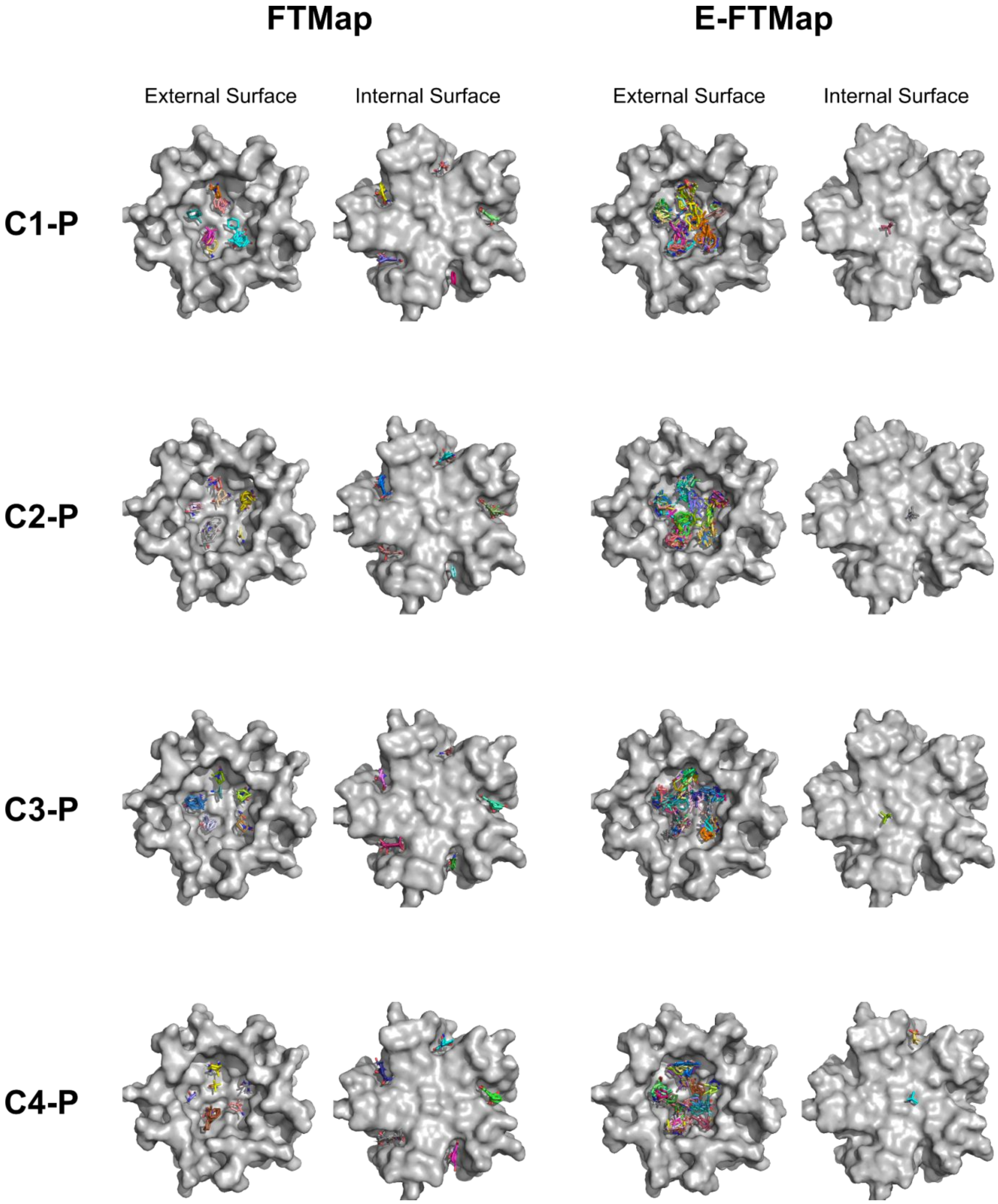

Support Information Figure S13. HCA and heatmaps displaying the similarity between pockets mapped with XDrugpy. a) trimer with chain differentiation; b) trimer without chain differentiation; c~~a~~) pentamer with chain differentiation; d~~b~~) pentamer without chain differentiation

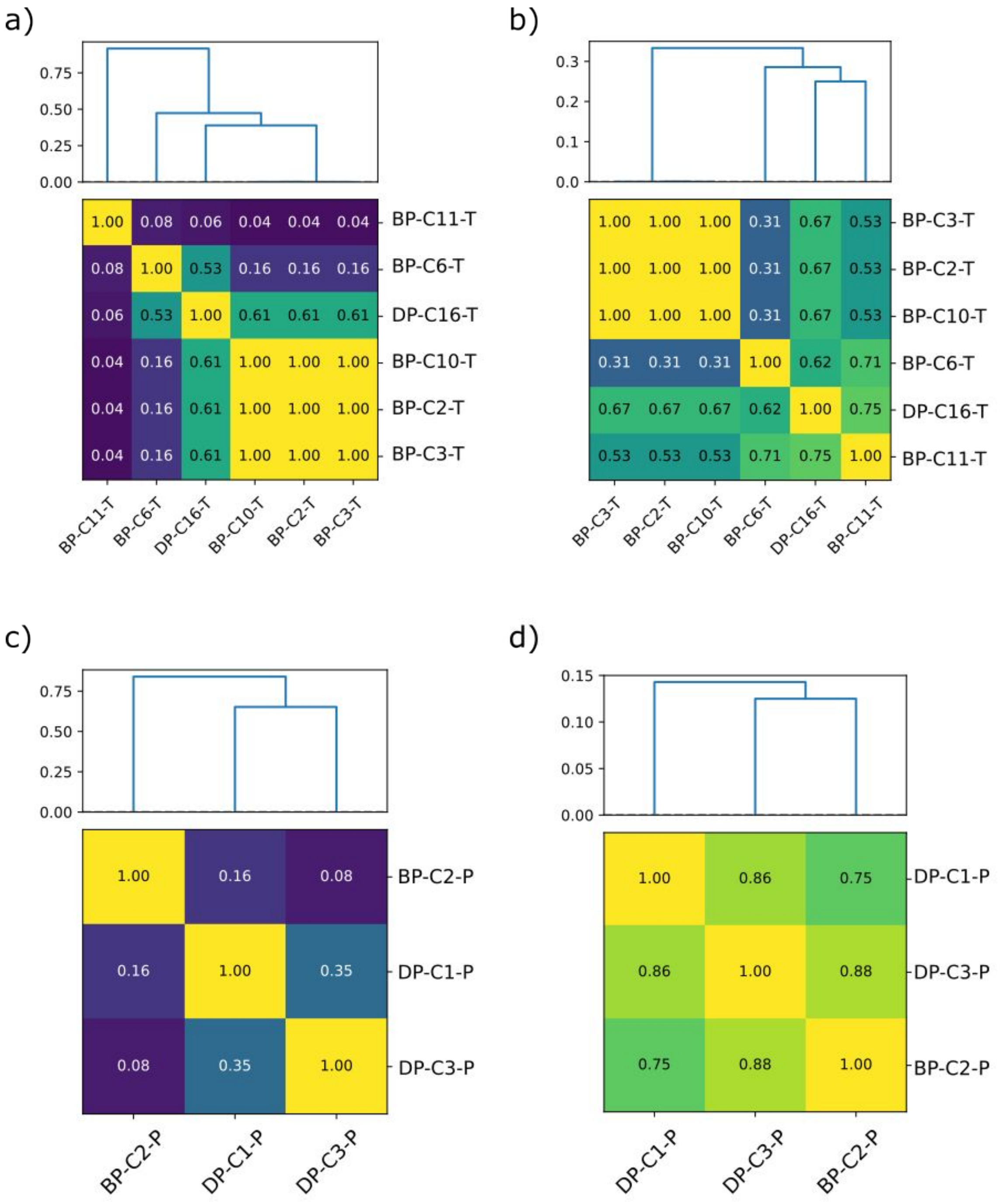

Support Information Figure S14. Interaction fingerprints derived from druggable and borderline druggable pockets identified by XDrugPy in the trimeric biological assembly.

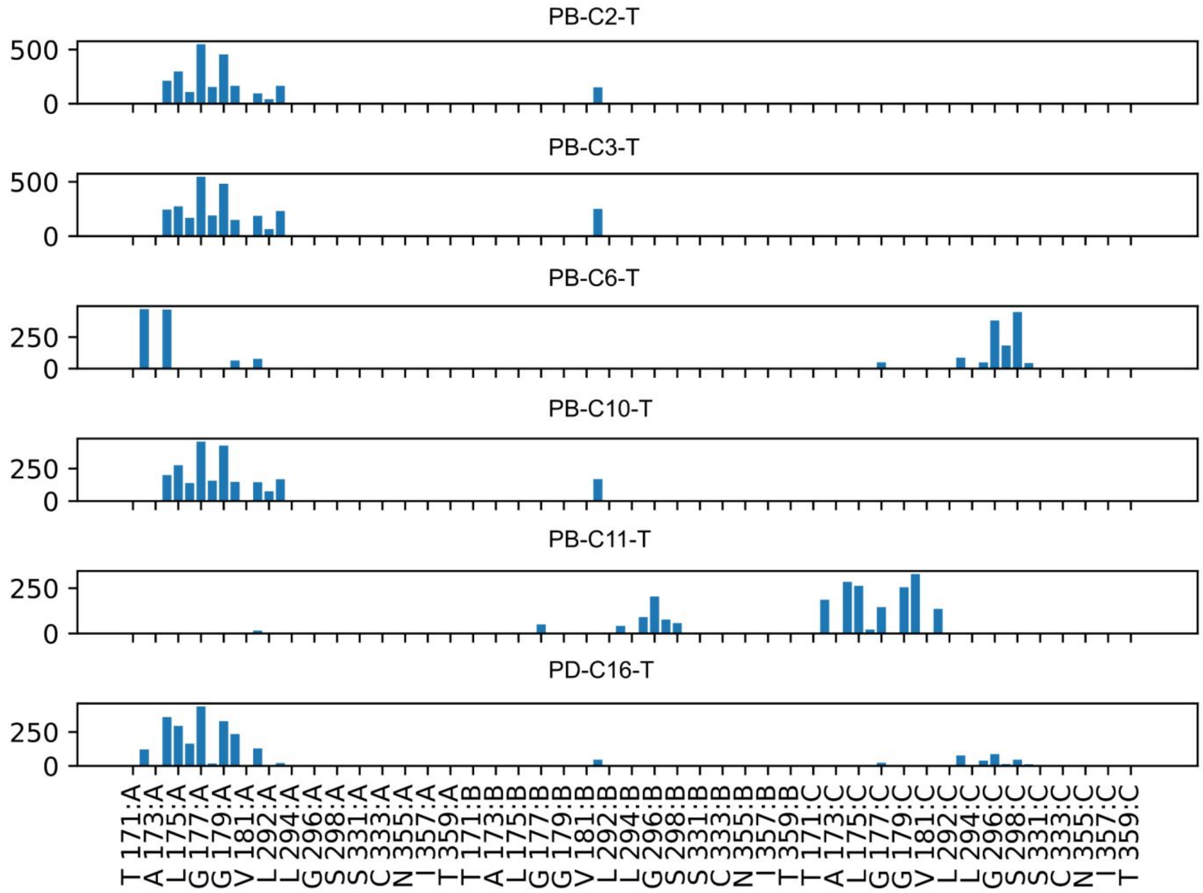

Support Information Figure S15. Interaction fingerprints derived from druggable and borderline druggable pockets identified by XDrugPy in the pentameric biological assembly.

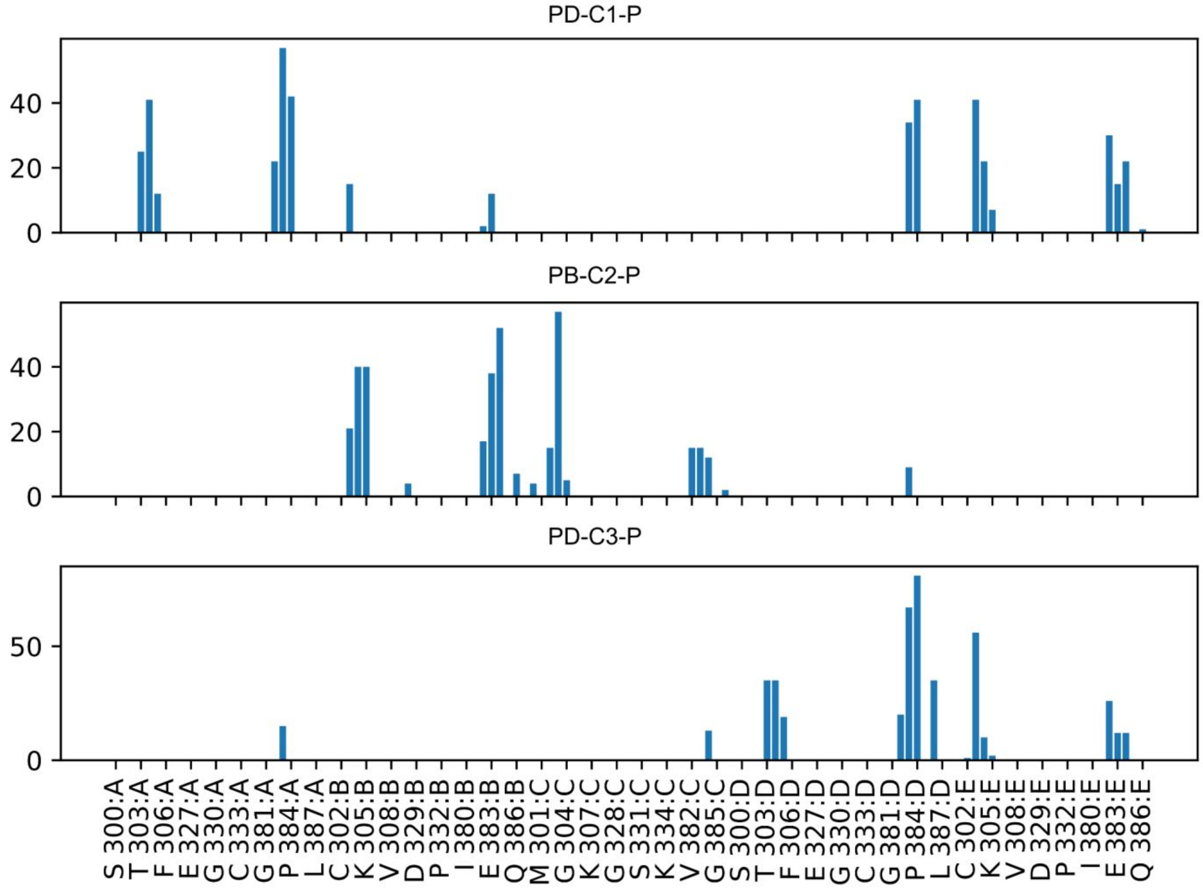

Support Information Figure S16. Sequence alignment of 32 sequences, among different DENV serotypes and other flaviviruses. The DENV2 reference sequence is highlighted in blue, and the pocket residues in the trimer are highlighted in red.

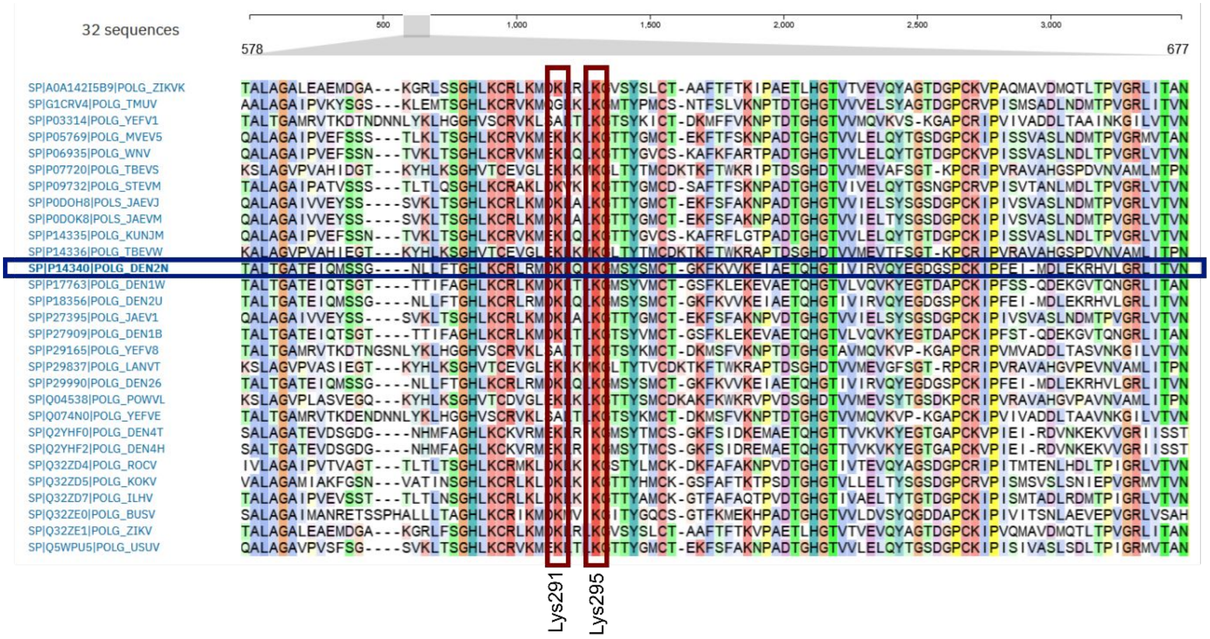

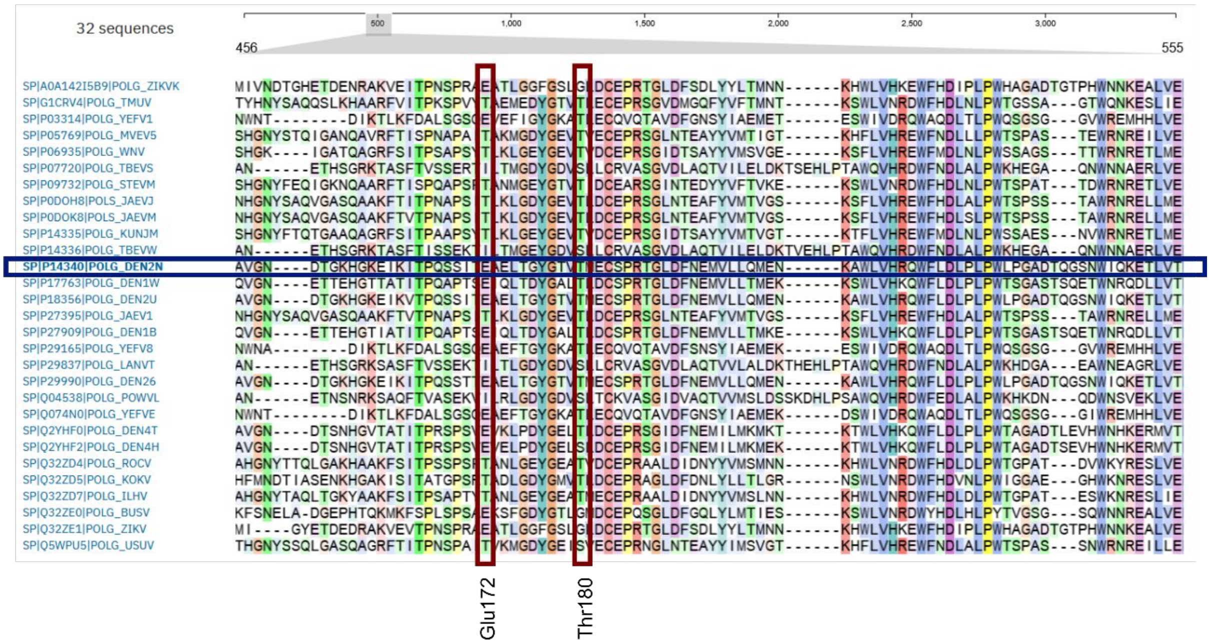

Support Information Figure S17. Sequence alignment of 32 sequences, among different DENV serotypes and other flaviviruses. The DENV2 reference sequence is highlighted in blue, and the pocket residues in the pentamer are highlighted in red.

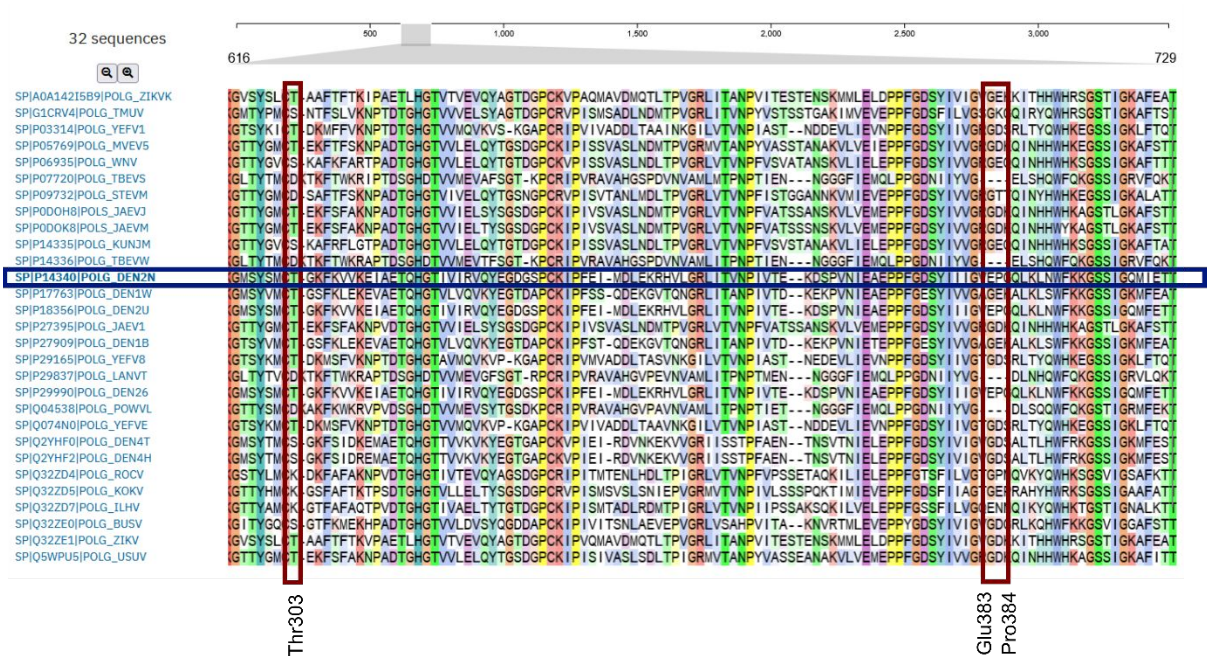

Support Information Figure S18. Structures of the trimeric and pentameric assemblies colored by sequence conservation score calculated with ChimeraX in three different multiple sequence alignments (MSA) performed with UniProt. Residues colored blue are less conserved, while residues colored red are more conserved. Proposed Pharmacophoric residues are shown as sticks. Alignment 1 was performed with 32 different flaviviruses sequences, alignment 2 with 29 sequences of the four DENV serotypes, and alignment 3 with 14 DENV-2 sequences. Consensus for the proposed pharmacophoric residues in each MSA is also shown. a) Conservation scores for alignment 1 projected on the trimeric assembly; b) Conservation scores for alignment 1 projected on the pentameric assembly; c) Conservation scores for alignment 2 projected on the trimeric assembly; d) Conservation scores for alignment 2 projected on the trimeric assembly; e) Conservation scores for alignment 3 projected on the trimeric assembly; f) Conservation scores for alignment 3 projected on the pentameric assembly.

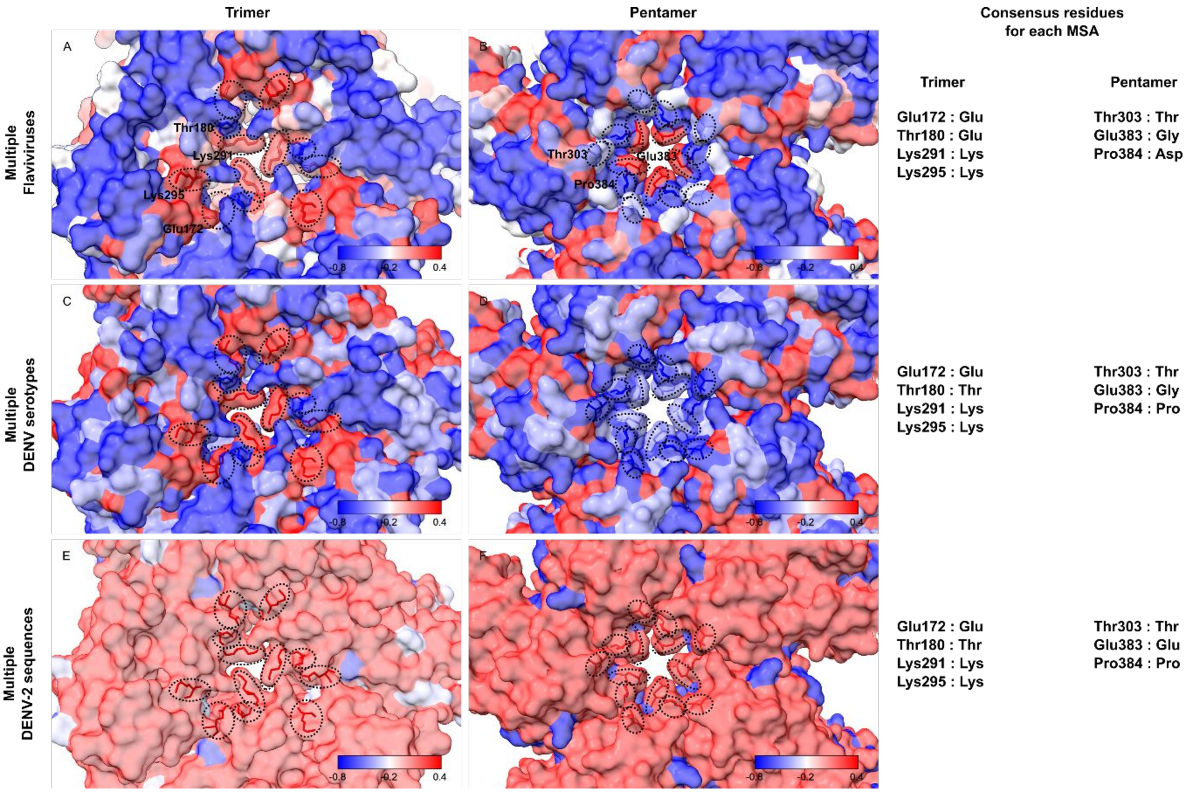

Support Information Table S1. Cryo-EM Structures deposited on PDB with similar assemblies to PDB ID 3J27.

PDB ID	Description	Resolution (Å)	Organism	Release Year	Structure Match Score	Oligomeric Count	Global Symmetry
3J27	CryoEM structure of Dengue virus	3.6	Denv-2	2012	100	360	Icosahedral - I
8Y3G	Cryo-EM structure of dengue virus serotype 2 strain D2Y98P-PP1 N153Q mutant at 4 deg C	2.7	Denv-2	2025	96.97	360	Icosahedral - I
8Y3J	Cryo-EM structure of dengue virus serotype 2 strain D2Y98P-PP1 at 4 deg C	3.2	Denv-2	2025	96.63	360	Icosahedral - I
3J6S	Cryo-EM structure of Dengue virus serotype 3 at 28 degrees C	6	Denv-3	2015	93.81	360	Icosahedral - I
5IRE	The cryo-EM structure of Zika Virus	3.8	Zika Virus	2016	93.3	360	Icosahedral - I
5IZ7	Cryo-EM structure of thermally stable Zika virus strain H/PF/2013	3.7	Zika Virus	2016	92.46	360	Icosahedral - I
7KV8	Chimeric flavivirus between Binjari virus and Dengue virus serotype-2	2.5	Denv-2 / Binjari Chimera	2020	92.28	360	Icosahedral - I
3J6T	Cryo-EM structure of Dengue virus serotype 3 at 37 degrees C	7	Denv-3	2015	91.26	360	Icosahedral - I
7KV9	Chimeric flavivirus between Binjari virus and West Nile (Kunjin) virus	2.9	West Nile / Binjari Chimera	2020	90.05	360	Icosahedral - I
7KVB	Chimeric flavivirus between Binjari virus and Murray Valley encephalitis virus	3.7	Murray Valley Encephalitis / Benjari Chimera	2020	88.98	360	Icosahedral - I
7KVA	Structure of West Nile virus (Kunjin)	3.1	West Nile Virus	2020	88.71	360	Icosahedral - I
4CBF	Near-atomic resolution cryo-EM structure of Dengue serotype 4 virus	4.1	Denv-4	2013	88	360	Icosahedral - I
4CCT	Dengue 1 cryo-EM reconstruction	4.5	Denv-1	2013	87.02	360	Icosahedral - I
6CO8	Structure of Zika virus at a resolution of 3.1 Angstrom	3.1	Zika Virus	2018	85.38	360	Icosahedral - I
7LCG	The mature Usutu SAAR-1776, Model A	2.42	Usutu Virus	2021	85.16	360	Icosahedral - I
7LCH	The mature Usutu SAAR-1776, Model B	2.35	Usutu Virus	2021	85.12	360	Icosahedral - I
6ZQV	Cryo-EM structure of mature Spondweni virus	2.6	Spondweni Virus	2021	84.76	360	Icosahedral - I
6ZQU	Cryo-EM structure of mature Dengue virus 2 at 3.1 angstrom resolution	3.1	Denv-2	2021	77.73	360	Icosahedral - I
5WSN	Structure of Japanese encephalitis virus	4.3	Japanese Encephalitis Virus	2017	73.86	360	Icosahedral - I
7ESD	Mature Donggang virus	3.9	Donggang Virus	2022	73.43	360	Icosahedral - I
5O6A	The cryo-EM structure of Tick-borne encephalitis virus mature particle	3.9	Tick-Borne Encephalitis Virus	2018	71.88	360	Icosahedral - I
7V3F	DENV2_NGC_Fab_C10 28degree (1Fab:3E)	3.1	Denv-2	2021	60.25	360	Icosahedral - I
9H28	Alternative conformation LGTV with TBEV prME	3.22	Tick-Borne Encephalitis Virus / Langat Virus	2024	59.94	240	Icosahedral - I
9FK0	LGTV with TBEV prME	3.22	Tick-Borne Encephalitis Virus / Langat Virus	2024	59.69	240	Icosahedral - I
9FOJ	LGTV TP21. Langat virus, strain TP21	3.82	Langat Virus	2024	58.91	6	Icosahedral - I
7Z51	Tick-borne encephalitis virus Kuutsalo-14	3.3	Tick-Borne Encephalitis Virus	2022	55.9	900	Icosahedral - I
1P58	Complex Organization of Dengue Virus Membrane Proteins as Revealed by 9.5 Angstrom Cryo-EM reconstruction	9.5	Denv-2	2003	54.4	360	Icosahedral - I

Support Information Table S2. FTMap results from mapping the representative prE biological assemblies (trimers highlighted in gray and pentamers in white). Mapping was focused on the GAG-binding pocket and the location of consensus sites (CS) on the exterior surface.

Frame	Number of CSs	CS0	CS1	CS2	CS3
C1	5	17	12	7	3
C2	5	15	13	6	5
C3	5	15	15	15	10
C4	6	16	13	10	5
C5	7	18	15	10	6
C6	6	17	15	14	6
C7	5	17	13	13	6
C8	4	18	15	12	2
C9	5	16	15	13	5
C10	7	18	15	6	6
C11	5	24	15	13	9
C12	6	15	13	12	7
C13	5	17	16	16	15
C14	6	18	14	10	4
C15	7	16	15	11	9
C16	7	18	17	12	6

Support Information Table S3. E-FTMap results from mapping the representative prE biological assemblies. Unlike FTMap, E-FTMap clusters probes by interaction type, in addition to coordinates. The number of probes in the highest-ranking cluster for each probe type is shown.

Frame	Number of CSs	C0 Halogen	C0 Aromatic	C0 Acceptor	C0 Donor	C0 Apolar
C1-T	45	8	30	33	42	42
C2-T	47	12	21	25	22	45
C3-T	46	10	21	24	27	36
C4-T	47	9	23	35	33	25
C5-T	49	10	23	29	28	41
C6-T	42	7	20	31	42	49
C7-T	44	14	25	18	42	39
C8-T	46	11	32	34	42	48
C9-T	40	10	30	39	52	58
C10-T	47	9	21	19	29	32
C11-T	46	10	27	27	33	44
C12-T	47	13	23	30	28	43
C13-T	47	10	32	33	26	45
C14-T	46	10	23	33	45	53
C15-T	47	10	24	32	41	34
C16-T	44	9	30	27	39	53
C1-P	37	12	27	4	34	47
C2-P	44	9	24	6	19	36
C3-P	35	10	23	7	41	31
C4-P	39	9	21	4	24	44

Support Information Table S4. The number of probe clusters identified by FTMap and E-FTMap located on the external and internal surfaces.

System	HCA Cluster	Structure Frame	Server	Number of Probe Clusters	
				External Surface	Internal Surface
**Pentamer**	1	2296	E-FTMap	37	1
			FTMap	6	5
	2	46	E-FTMap	44	1
			FTMap	6	5
	3	626	E-FTMap	35	1
			FTMap	6	5
	4	119	E-FTMap	39	2
			FTMap	5	5
**Trimer**	1	698	E-FTMap	45	1
			FTMap	5	7
	2	1255	E-FTMap	47	3
			FTMap	5	7
	3	1024	E-FTMap	46	1
			FTMap	5	8
	4	2070	E-FTMap	47	1
			FTMap	6	5
	5	1039	E-FTMap	49	0
			FTMap	7	5
	6	1501	E-FTMap	42	3
			FTMap	5	6
	7	2256	E-FTMap	44	2
			FTMap	5	8
	8	2399	E-FTMap	46	0
			FTMap	4	7
	9	660	E-FTMap	40	3
			FTMap	5	8
	10	1026	E-FTMap	47	0
			FTMap	7	5
	11	751	E-FTMap	46	1
			FTMap	5	5
	12	16	E-FTMap	47	3
			FTMap	6	5
	13	1	E-FTMap	47	0
			FTMap	5	4
	14	223	E-FTMap	46	0
			FTMap	6	5
	15	308	E-FTMap	47	1
			FTMap	7	5
	16	153	E-FTMap	44	0
			FTMap	6	5
